# A unified mediation analysis framework for integrative cancer proteogenomics with clinical outcomes

**DOI:** 10.1093/bioinformatics/btad023

**Published:** 2023-01-17

**Authors:** Licai Huang, James P Long, Ehsan Irajizad, James D Doecke, Kim-Anh Do, Min Jin Ha

**Affiliations:** Department of Biostatistics, The University of Texas MD Anderson Cancer Center, Houston, TX, USA; Department of Biostatistics, The University of Texas MD Anderson Cancer Center, Houston, TX, USA; CSIRO, Royal Brisbane and Women’s Hospital, Brisbane, Australia; Department of Biostatistics, The University of Texas MD Anderson Cancer Center, Houston, TX, USA; Department of Health Informatics and Biostatistics, Graduate School of Public Health, Yonsei University, Seoul, South Korea

## Abstract

**Motivation:**

Multilevel molecular profiling of tumors and the integrative analysis with clinical outcomes have enabled a deeper characterization of cancer treatment. Mediation analysis has emerged as a promising statistical tool to identify and quantify the intermediate mechanisms by which a gene affects an outcome. However, existing methods lack a unified approach to handle various types of outcome variables, making them unsuitable for high-throughput molecular profiling data with highly interconnected variables.

**Results:**

We develop a general mediation analysis framework for proteogenomic data that include multiple exposures, multivariate mediators on various scales of effects as appropriate for continuous, binary and survival outcomes. Our estimation method avoids imposing constraints on model parameters such as the rare disease assumption, while accommodating multiple exposures and high-dimensional mediators. We compare our approach to other methods in extensive simulation studies at a range of sample sizes, disease prevalence and number of false mediators. Using kidney renal clear cell carcinoma proteogenomic data, we identify genes that are mediated by proteins and the underlying mechanisms on various survival outcomes that capture short- and long-term disease-specific clinical characteristics.

**Availability and implementation:**

Software is made available in an R package (https://github.com/longjp/mediateR).

**Supplementary information:**

Supplementary data are available at *Bioinformatics* online.

## 1 Introduction

One of the main goals in cancer research is to develop accurate prognostic models that can stratify patients into risk groups and suggest customized therapeutic strategies (Yuan *et al.*, 2014). Genomic and transcriptomic profiling and the association analysis with clinical outcomes such as patients’ survival times have greatly improved the understanding of the clinical importance of a given gene (Tang *et al.*, 2017) or group of biologically related genes, called meta-genes (Alcaraz *et al.*, 2017). Proteins, however, represent the downstream effect of changes that are accumulated at the DNA and mRNA levels and the effects of genomic and trancriptomic changes on a phenotype such as survival may be mediated by changes in protein expression (Kumar *et al.*, 2016). Proteogenomics is a field of research that integrate genomics, transcriptomics and proteomics to aid the protein-level understanding of gene expression and to help refine gene models (Nesvizhskii, 2014). Connecting tumor-derived DNA, RNA and protein measurements into a central-dogma perspective has the potential to improve clinical characterization and treatment for patients with cancer (Rodriguez *et al.*, 2021).

The rich data source from The Cancer Genome Atlas (TCGA) project has excelled prognostic modeling of multilevel molecular profiles to patients’ clinical outcomes. Overall survival (OS) is the most well-defined clinical endpoint in TCGA studies with an event as death from any cause. However, this endpoint assesses the long-term outcome and may not reflect disease-specific biology due to inclusion of non-cancer causes. To this end, the TCGA Pan-Cancer Clinical Data Resource (TCGA-CDR) generated standardized clinical outcome endpoints including OS, disease-specific survival (DSS) and progression-free interval (PFI), of more than 11 000 human tumors across 33 different cancer types and subtypes (Liu *et al.*, 2018). DSS captures patients’ long-term survival outcome which is disease specific while PFI reflects short-term clinical behavior because patients generally develop disease recurrence or progression before dying.

Our study is motivated by kidney renal clear cell carcinoma (KIRC) which is the most common and lethal type of kidney cancer. TCGA Research Network (2013) identified prognostic signatures on OS at the levels of mRNA, miRNA, DNA methylation and proteins that are involved in a metabolic shift for aggressive tumors, including the phosphatase and tensin homolog (PTEN) gene, the citrate (TCA) cycle, fatty acid synthesis (FAS), AMP-activated kinase (AMPK) complex and the pentose phosphate pathway. Most TCGA studies including the KIRC study concatenate the multilevel features into a vector at a single level without consideration of regulatory information flow that occurs cross-platform. Modeling multiplatform data in an ordered domain following the central-dogma perspective allows for regulations from gene to protein expressions and to a clinical outcome. Our overarching goal is to investigate how changes in gene expressions in key pathways change the clinical outcomes through altering the expression levels of multiple proteins in a major function space in cancer. For our specific example of TCGA KIRC, we use the curated and filtered survival endpoints that recapitulate patients’ short- and long-term disease-specific clinical responses from TCGA-CDR. Identification and quantification of such protein mediators enhance scientific understanding of how changes at the mRNA level impact a phenotype, which will further facilitate the development of novel diagnostic and therapeutic strategies. In another case study, we also evaluate mutation–survival relations. When a mutation occurs, the downstream protein products may be altered. Identifying proteins that mediate the mutation’s effect on survival sheds light on potential targets for therapeutic intervention in tumors carrying such a mutation. For these scientific questions, we propose a general mediation framework for various types of outcome variables, including without limitation to time-to-event outcomes.

The literature on mediation analysis dates back to Baron and Kenny (1986), who studied the concept in linear models with a single mediator. Robins and Greenland (1992) and Pearl (2001) generalized the definitions of direct and indirect effects to include non-linear models. Since then, estimation of mediation effects has been studied with various outcome distributions (Imai *et al.*, 2010a), with multiple mediators (Fasanelli *et al.*, 2019; Huang and Pan, 2016; Zhao *et al.*, 2020) and on different effect scales (VanderWeele and Vansteelandt, 2010). These models for binary outcomes, however, require the response to be rare with logistic outcome models (Huang *et al.*, 2014; VanderWeele and Vansteelandt, 2010). Along the same line, Gaynor *et al.* (2018) proposed a probit approximation to the logistic function, designed for common responses where the rare disease assumption does not hold.

For survival responses, Tein and MacKinnon (2003) considered both proportional hazard models and an accelerated failure time model for mediation analysis. They quantified the mediation effect based on both mean difference scale and the product of the regression coefficients, but this approach does not have a clear causal interpretation. Using the counterfactual approach, VanderWeele (2011) derived a proportional hazards model with a rare outcome or accelerated failure time model generally on the mean difference scale. However, the former model requires a rare outcome and the latter requires strong parametric assumptions. In addition, these literatures can only accommodate a single mediator. Fasanelli *et al.* (2019) proposed mediation analysis for survival outcomes through specifications of a response model and a propensity model, using inverse probability weighting to estimate the causal effects on the hazard ratio scale. This approach avoids specification of a model for mediators but can only accommodate a binary or categorical exposure. In the presence of high-dimensional mediators, Zhang *et al.* (2021) and Rijnhart *et al.* (2021) proposed a regularized Cox proportional hazard model on the pathway of mediators to outcome. They quantified the mediation effect based on the product of the regression coefficients and the effect on difference of log-hazard scale, respectively. However, the former work does not provide quantification for direct/indirect effects and the latter requires a rare outcome. In addition, both do not fit in with multiple exposures. Other frameworks, such as Imai *et al.* (2010a), accommodate a wide range of response models but measure effects only on the mean difference scale, which is often not appropriate for binary or survival responses. With censored survival times, the mean survival time may not be identifiable. To this end, we consider the difference of restricted mean lifetime, which is more interpretable (Chen and Tsiatis, 2001).

In this article, we develop a unified mediation analysis framework for multiomics and clinical datasets in cancer, which contain multiple potential causes, multiple mediators and categorical and survival responses that are not suitable for linear models, as well as the continuous type. Our novel contributions are summarized as follows: our framework (1) estimates mediation effects with multiple mediators that may form a correlation structure without requiring specification of the causal structure among mediators; (2) handles continuous, binary and survival response models while measuring mediation effect on various scales appropriate to the given response model; (3) eliminates restrictive assumptions such as requiring binary exposures or ‘rare diseases’ for binary response models and (4) models high-dimensional mediators by employing regularized outcome models with ridge penalties, which are seamlessly incorporated in the effect estimation procedure and the resampling-based inferential procedure. A publicly available *mediateR* package incorporating all of this functionality facilitates wide use of this framework by others.

This work is organized as follows: Section 2 describes our framework in-depth. The value of our framework is demonstrated in simulations in Section 3 and an application to the TCGA KIRC dataset in Section 4. We conclude with a discussion in Section 5.

## 2 Causal mediation analysis framework

### 2.1 Causal structure

**Fig. 1.**
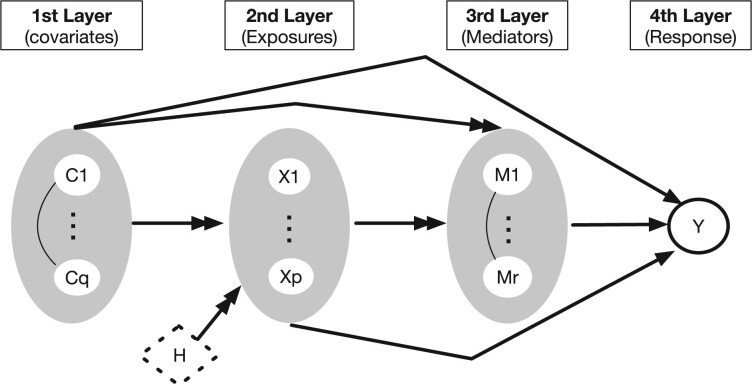
Illustration of directed acyclic graph for mediation analysis where four disjoint sets of variables (nodes), covariates (C), exposures (X), mediators ( ) and response (Y) have their unique order, C<X<M<Y. The goal is to assess the causal impact of changing any single exposure $X\in \{X_{1},\ldots X_{p}\}$ on an outcome *Y* and quantify how much of this effect is mediated by the set of mediators $M=\{M_{1},\ldots ,M_{r}\}$. The variables $C=\{C_{1},\ldots ,C_{q}\}$ represent potential confounders. Our model assumes that the causal agents may be linked by unobserved factors (H) and permits mediators to have internal causal or correlation structure

Our mediation analysis framework is based on the multilayered graphical structure illustrated in Figure 1. We consider q clinical variable $C={\left(C_{1},\ldots ,C_{q}\right)}^{T}\in R^{q}$ as covariates, p genes $X={\left(X_{1},\ldots ,X_{p}\right)}^{T}\in R^{p}$ as exposures, r proteins $M={\left(M_{1},\ldots ,M_{r}\right)}^{T}\in R^{r}$ as mediators, and a survival outcome Y as response. Variables in each layer (e.g. X) potentially have causal influence on variables in the downstream layers (e.g. M and Y). Our framework assesses the causal effect of any of the $X_{i}$ for i=1,…,p on the outcome Y and quantifies how much of this effect passes through the set of mediators M, termed indirect effect, and how much of the effect is through other mechanisms, termed direct effect (Pearl, 2001, 2009).

We assume that the correlations among the exposures are the result of observed confounders C, which may also confound the mediator–exposure relation and unobserved confounders H that only causally influence X. Thus, the causal assumptions imply $X_{i}⊥X_{j}|C,H$. Note that the mediation effects for *X_i_* in our model cannot be derived by treating the other X variables (termed $X_{-i}$) as confounders since this would assume that $X_{-i}$ are causes of $X_{I}$. Instead, by allowing H, effects are identified without specifying the causal ordering of the exposures X. In the real data example (Section 4), we investigate the effects of a gene $X_{I}$ within coordinating modules X such as functional or cell signaling pathways that are relevant to metabolic process on survival Y and the extent to which this effect is mediated by proteins involved in major function spaces in cancer M (translational mechanism as indirect effect) or occurs through unmeasured gene regulatory paths of X to the outcome (direct effect). The latent factors H induce correlations among X as oncogenes and tumor suppressor genes operate within pathways and functional networks (Boehm and Hahn, 2011).

In the context of our real data application in Section 4, for each gene $X_{i}$, we calibrate a joint indirect effect for the set of proteins M (mediators), rather than attempting to assess the indirect effect of individual protein $M_{j}$. There are several reasons and implications for this approach. We avoid having to specify any internal causal structure among the proteins M as the causal structure is not identifiable from observational data [see Akbani *et al.* (2014) for experimental design of reverse-phase protein array (RPPA) data]. Moreover, path-specific effects of $X_{i}$ on Y through only $M_{j}$ cannot be identified when there is a mediator–outcome confounder, which is itself influenced by the exposure $X_{j}$ (Avin *et al.*, 2005; VanderWeele *et al.*, 2014). This is likely to be the case when the proteins M represent a set of multivariate measures on the same platform where a high level of interactions is present owing to protein–protein interactions (Szklarczyk *et al.*, 2021). Thus, the indirect effect of our analysis is the causal effect of a gene $X_{i}$ on Y that is jointly mediated by the entire functional proteomic space that encompasses key functional and signaling pathways of human cancer (Akbani *et al.*, 2014).

### 2.2 Direct and indirect effects

Counterfactual random variables are used to formally define causal interventions and the notions of direct and indirect effects. Let $Y^{X=x\prime }$ be the value of Y obtained by setting X=x′, possibly counter to fact. For notational simplicity, we will write $Y^{x\prime }$ when it is clear that X is being set to x′. Counterfactual notation can also express interventions on multiple variables. For example, $Y^{x\prime ,m\prime }$ is the value Y would obtain by setting X=x′ and M=m′. Direct and indirect effects are represented as functions of nested counterfactual variables, such as $Y^{x\prime \prime ,M^{x\prime }}$, the value Y would have obtained had X been set to x″ and had M been set to the value it would have obtained had *x* been set to x′.

We review the terms natural direct effect, natural indirect effect and total effect as used in VanderWeele and Vansteelandt (2010) and Tchetgen and Shpitser (2012). These definitions admit a decomposition of total effect into indirect (effect of X on Y passing through M) and direct (effect of X on Y not through M) effects. We analyze these on the mean difference scale, the odds scale and the restricted mean difference scale for continuous, binary and survival outcomes, respectively. Counterfactual independence and consistency assumptions in Supplementary Section S1 are needed to derive direct and indirect effects in terms of the joint distribution. The proofs of the following results can be found in Supplementary Section S2 and estimators of these quantities are discussed in Section 2.4.

#### 2.2.1 Mean difference and restricted mean difference scale

The average direct effect on the mean difference scale when changing $X_{i}$ from x′ to x″ with respect to mediators M is defined as

(1) $${\text{DE}}_{X_{i}}(x\prime ,x\prime \prime )=E[Y^{X_{i}=x\prime \prime ,M^{X_{i}=x\prime }}-Y^{X_{i}=x\prime }].$$

The counterfactual random variable $Y^{X_{i}=x\prime \prime ,M^{X_{i}=x\prime }}$ is the value Y would have obtained had $X_{i}$ been set to the value x″ and had M been set to the value it would have obtained had $X_{i}$ been set to x′. In contrast, $Y^{X_{i}=x\prime }$ is the value of Y when $X_{i}$ is set to x′ (Note $Y^{X_{i}=x\prime }=Y^{X_{i}=x\prime ,M^{X_{i}=x\prime }}$ by Assumption 7 in Supplementary Section S1). Thus, the difference in these counterfactual quantities captures the intuitive notion of the change in Y when the direct link from $X_{i}$ to Y is changed from x′ to x″ but the indirect link (through M) remains at x′. Similarly, the natural indirect effect is defined as

$${\text{IE}}_{X_{i}}(x\prime ,x\prime \prime )=E[Y^{X_{i}=x\prime \prime }-Y^{X_{i}=x\prime \prime ,M^{X_{i}=x\prime }}].$$

Finally, we have the general mediation formula

$${\text{TE}}_{X_{i}}(x\prime ,x\prime \prime )={\text{DE}}_{X_{i}}(x\prime ,x\prime \prime )+{\text{IE}}_{X_{i}}(x\prime ,x\prime \prime ).$$

With survival outcomes, estimators of the expected response (i.e. E[Y|x,m,c]) often have high variance in the presence of censoring. Instead, we consider mean survival time restricted to a fixed time L, that is, E[min(Y,L)|x,m,c] (Chen and Tsiatis, 2001). The restricted mean is interpreted as the population average of the amount of survival time experienced during the initial L time of follow-up, providing an interpretable and clinically meaningful summary of the survival in the presence of censoring (Uno *et al.*, 2014). Similar definitions of direct, indirect and total effects can be applied to the restricted mean survival scale, denoted with superscript (${}^{R}$),

$$\begin{array}{c} {\text{DE}}_{X_{i}}^{R}(x\prime ,x\prime \prime )=E[\min(Y^{X_{i}=x\prime \prime ,M^{X_{i}=x\prime }},L)-\min(Y^{X_{i}=x\prime },L)],\\ {\text{IE}}_{X_{i}}^{R}(x\prime ,x\prime \prime )=E[\text{min}(Y^{X_{i}=x\prime \prime },L)-\text{min}(Y^{X_{i}=x\prime \prime ,M^{X_{i}=x\prime }},L)],\\ {\text{TE}}_{X_{i}}^{R}(x\prime ,x\prime \prime )={\text{DE}}_{X_{i}}^{R}(x\prime ,x\prime \prime )+{\text{IE}}_{X_{i}}^{R}(x\prime ,x\prime \prime ). \end{array}$$

The left-hand side of Equation (1) cannot be directly estimated because it depends on counterfactual random variables that are not observed. However, it is possible to express the direct effect as a function of the joint distribution of observed random variables, which then facilitates estimation. Let $g:R^{1}\to R^{1}$. In the mean difference scale or odds scale, g(·)=·, and in the restricted mean difference scale, denoted with superscript (${}^{R}$), g(·)=min(·,L). We derive the ‘direct effect on the (restricted) mean difference’ as

(2) $${\text{DE}}_{X_{i}}(x\prime ,x\prime \prime )\;\text{or}\;{\text{DE}}_{X_{i}}^{R}(x\prime ,x\prime \prime )=\underset{\equiv e(x\prime ,x\prime \prime )\;\text{or}\;e^{R}(x\prime ,x\prime \prime )}{\underset{︸}{\int E[g(Y)|x\prime \prime ,x_{-i},m,c]p(m|x\prime ,x_{-i},c)p(x_{-i},c)dx_{-i}dmdc}},-\underset{\equiv e(x\prime ,x\prime )\;\text{or}\;e^{R}(x\prime ,x\prime )}{\underset{︸}{\int E[g(Y)|x\prime ,x_{-i},m,c]p(m|x\prime ,x_{-i},c)p(x_{-i},c)dx_{-i}dmdc}}.$$

Again the natural indirect effect can be represented in terms of the joint probability distribution of the observed random variables. We derive the indirect effect on the (restricted) mean difference as

$${\text{IE}}_{X_{i}}(x\prime ,x\prime \prime )\;\text{or}\;{\text{IE}}_{X_{i}}^{R}(x\prime ,x\prime \prime )=$$

$$\underset{\equiv e(x\prime \prime ,x\prime \prime )\;\text{or}\;e^{R}(x\prime \prime ,x\prime \prime )}{\underset{︸}{\int E[g(Y)|x\prime \prime ,x_{-i},m,c]p(m|x\prime \prime ,x_{-i},c)p(x_{-i},c)dx_{-i}dmdc}},$$

$$-\underset{\equiv e(x\prime ,x\prime \prime )\;\text{or}\;e^{R}(x\prime ,x\prime \prime )}{\underset{︸}{\int E[g(Y)|x\prime \prime ,x_{-i},m,c]p(m|x\prime ,x_{-i},c)p(x_{-i},c)dx_{-i}dmdc}}.$$

The mediation formula states that the total effect is the sum of the direct and indirect effects. The relative contributions of the direct and the indirect effects are important for understanding the paths by which $X_{i}$ causes changes in Y. For example, if there is no direct effect, then all changes in Y caused by $X_{i}$ pass through M.

#### 2.2.2 Odds scale

The total, direct and indirect effects require computing three quantities, e(x″,x″),e(x′,x″),e(x′,x′). For binary outcome y, VanderWeele and Vansteelandt (2010) defined direct and indirect effects on the odds scale:

$${\text{DE}}^{o}(x\prime ,x\prime \prime )=\frac{\frac{e(x\prime \prime ,x\prime \prime )}{1-e(x\prime \prime ,x\prime \prime )}}{\frac{e(x\prime ,x\prime \prime )}{1-e(x\prime ,x\prime \prime )}},\;{\text{IE}}^{o}(x\prime ,x\prime \prime )=\frac{\frac{e(x\prime ,x\prime \prime )}{1-e(x\prime ,x\prime \prime )}}{\frac{e(x\prime ,x\prime )}{1-e(x\prime ,x\prime )}}.$$

The total is defined as ${\text{TE}}^{o}(x\prime ,x\prime \prime )={\text{DE}}^{o}(x\prime ,x\prime \prime ){\text{IE}}^{o}(x\prime ,x\prime \prime ).$

### 2.3 Probability models

The probabilistic relationships among the variables in Figure 1 are specified with parametric and semi-parametric statistical models for any configurations c, x, m, and *y* of C, X, M and Y, respectively. We assume linear relations for the conditional distribution of M given X and C. Specifically,

(3) $$m=\beta^{\left(X\right)}x+\beta^{\left(C\right)}c+\beta^{\left(0\right)}+\epsilon ,$$

where $\beta^{\left(X\right)}=(\beta_{j,i}^{\left(X\right)})\in R^{r\times p}$, $\beta^{\left(C\right)}=(\beta_{j,i}^{\left(C\right)})\in R^{r\times q},\;\beta^{\left(0\right)}=(\beta_{j}^{\left(0\right)})\in R^{r},\;\epsilon ∼N_{r}(0,\Sigma_{\epsilon })$ and $\Sigma_{\epsilon }\in R^{r\times r}$ is a covariance matrix. In the case where mediators are conditionally independent given X and C, $\Sigma_{\epsilon }$ will be a diagonal matrix.

We consider three parametric models, linear, logistic and Cox proportional hazards, for linking Y with X, M and C for continuous, binary and time-to-event outcomes, respectively. Each of these models has parameters $\alpha =(\alpha^{\left(X\right)},\alpha^{\left(M\right)},\alpha^{\left(C\right)})$, where $\alpha^{\left(X\right)}=(\alpha_{j}^{\left(X\right)})\in R^{p}$, $\alpha^{\left(M\right)}=(\alpha_{j}^{\left(M\right)})\in R^{r}$, $\alpha^{\left(C\right)}=(\alpha_{j}^{\left(C\right)})\in R^{q}$ and $\alpha^{\left(0\right)}\in R^{1}$.

(4) $$\text{Linear:}\;y=x^{T}\alpha^{\left(X\right)}+m^{T}\alpha^{\left(M\right)}+c^{T}\alpha^{\left(C\right)}+\alpha^{\left(0\right)}+\delta ,$$

where $\delta ∼N(0,\sigma_{\delta }^{2})$ is independent of all other terms in the model.

(5) $$\begin{array}{l} \text{Logistic:}Y∼\text{Bernoulli}(p),\;\\ \text{logit}(p)=x^{T}\alpha^{\left(X\right)}+m^{T}\alpha^{\left(M\right)}+c^{T}\alpha^{\left(C\right)}+\alpha^{\left(0\right)}, \end{array}$$

Cox proportional hazards: The failure time Y is assumed to follow a hazard function model:

(6) $$h(y|x,m,c)=h_{0}(y)e^{x^{T}\alpha^{\left(X\right)}+m^{T}\alpha^{\left(m\right)}+c^{T}\alpha^{\left(C\right)}},$$

where $h_{0}$ is the unspecified baseline hazard.

### 2.4 Estimation and computation of effects

For linear models, the direct, indirect and total effects have simple definitions in terms of path coefficients from the probability models in Section 2.3. For non-linear models, we estimate model coefficients and then numerically approximate indirect and direct effect integrals in expressions derived in Section 2.2. In high-dimensional settings, regularized linear, logistic and Cox proportional hazards models with ridge penalties are incorporated in the model fitting steps to estimate the path coefficients. The tuning parameters were selected using cross-validation, minimizing the mean-squared error (Friedman *et al.*, 2010; Simon *et al.*, 2011). In the forthcoming subsections, we describe estimations of direct and indirect effects that are applicable to both non-regularized and regularized parameter estimates by numerical integration.

#### 2.4.1 Mean difference and odds scale

Both the mean difference and odds scale require estimates of three quantities: e(x″,x″),e(x′,x″),e(x′,x′). We discuss estimation of e(x′,x″). The algorithms for e(x″,x″) and e(x′,x′) are nearly identical. Recall

$$\begin{array}{c} e(x\prime ,x\prime \prime )\equiv \int E[Y|X_{i}=x\prime \prime ,x_{-i},m,c]p(m|X_{i}=x\prime ,x_{-i},c)\\ \times p(x_{-i},c)dx_{-i}dmdc. \end{array}$$

We plug estimates into unknown quantities in the integrand and use Monte Carlo sampling to approximate the integral. The quantity $p(m|X_{i}=x\prime ,x_{-i},c)p(x_{-i},c)$ is a distribution on $m,x_{-i},c$. We use the observed data samples $x_{-i,l}$ and $c_{l}$ for l=1,…,n as a draw from $p(x_{-i},c)$. We then draw ${\bar{m}}_{l}∼\hat{p}(m|X_{i}=x\prime ,x_{-i,l},c_{l})$. The bar in ${\bar{m}}_{l}$ denotes the fact that this is the data we simulate, not the actual observed mediator for sample l. The Monte Carlo approximation is

$$\hat{e}(x\prime ,x\prime \prime )=\frac{1}{n}\sum_{l=1}^{n}\hat{E}[Y|X_{i}=x\prime \prime ,x_{-i,l},{\bar{m}}_{l},c_{l}].$$

We specify estimates $\hat{E}[Y|x,m,c]$ using response models in Equations (4) and (5)

$$\begin{array}{c} \text{Linear:}\;\;\hat{E}[Y|x,m,c]=x^{T}{\hat{\alpha }}^{\left(X\right)}+m^{T}{\hat{\alpha }}^{\left(M\right)}+c^{T}{\hat{\alpha }}^{\left(C\right)}+{\hat{\alpha }}^{\left(0\right)},\\ \text{Logistic:}\;\hat{E}[Y|x,m,c]=\hat{p}(Y=1|x,m,c)\\ \;\;\;\;=\frac{1}{1+e^{-x^{T}{\hat{\alpha }}^{\left(X\right)}-m^{T}{\hat{\alpha }}^{\left(M\right)}-c^{T}{\hat{\alpha }}^{\left(C\right)}-{\hat{\alpha }}^{\left(0\right)}}}. \end{array}$$

For $\hat{p}(m|x,c)$, recall by Equation (3) that

$$m|x,c∼N(\beta^{\left(X\right)}x+\beta^{\left(C\right)}c+\beta^{\left(0\right)},\Sigma_{\epsilon }).$$

The ${\bar{m}}_{l}$ are simulated from the plug-in-based measure $\hat{p}(m|X_{i}=x\prime ,x_{-i,l},c_{l})$. We estimate $\Sigma_{\epsilon }$ using the sample covariance of the regression residuals $r_{l}=m_{l}-({\hat{\beta }}^{\left(X\right)}x_{l}+{\hat{\beta }}^{\left(C\right)}c_{l}+{\hat{\beta }}^{\left(0\right)})$.

#### 2.4.2 Restricted mean scale

On the restricted mean scale, the quantities of interest are $e^{R}(x\prime \prime ,x\prime \prime ),\;e^{R}(x\prime ,x\prime \prime ),\;e^{R}(x\prime ,x\prime )$. These are nearly identical to the terms for mean difference and odds scales with the exception that Y is replaced by min(Y,L) within the expectation. Thus, the numerical approximation to the integral follows the procedure in Section 2.4.1. The numerical approximation to the integral can be accomplished by deriving estimates for the survival function S(y|x,m,c)=P(Y>y|x,m,c). An estimator for the restricted mean is

$$\hat{E}[\text{min}(Y,L)|x,m,c]=\int_{0}^{L}\hat{S}(y|x,m,c)dy,$$

with estimates from the Cox proportional hazards model in Equation (6)

$$\hat{S}(y|x,m,c)=e^{\left(-\int_{0}^{y}{\hat{h}}_{0}(t)dt\right)e^{x^{T}{\hat{\alpha }}^{\left(X\right)}+m^{T}{\hat{\alpha }}^{\left(M\right)}+c^{T}{\hat{\alpha }}^{\left(C\right)}}},$$

where $\hat{h}$ is an estimate of the baseline hazard function and ${\hat{\alpha }}^{\left(X\right)},{\hat{\alpha }}^{\left(M\right)},{\hat{\alpha }}^{\left(C\right)}$ are coefficient estimates.

### 2.5 Bootstrap confidence intervals and hypothesis tests

There are several existing approaches for creating confidence intervals and performing hypothesis tests in mediation analysis. The problem of hypothesis testing for the existence of an indirect effect has generated particular interest because it is practically important and challenging, due to the composite nature of the null hypothesis (Barfield *et al.*, 2017). In univariate linear models, the null hypothesis of no indirect effect is $H_{0}:\beta^{\left(X\right)}\alpha^{\left(M\right)}=0$. Thus, the null can be true if either there is no exposure–mediator causal effect or if there is no mediator–response causal effect. Delta method-based approximations to the sampling distribution are not valid due to the non-normality of ${\hat{\beta }}^{\left(X\right)}{\hat{\alpha }}^{\left(M\right)}$ under the null hypothesis. The joint significance test proposes computing *P*-values for the tests $H_{0}:\beta^{\left(X\right)}=0$ and $H_{0}:\alpha^{\left(M\right)}=0$. The maximum of these *P*-values controls type I errors. This control is conservative in the case where both the exposure–mediator and mediator–response relations are null, that is, $\beta^{\left(x\right)}=\alpha^{\left(m\right)}=0$.

We propose computing confidence intervals and hypothesis tests using bootstrap sampling quantiles. Suppose *B* bootstrap samples of the data are taken. Let ${\hat{\text{IE}}}_{X_{i}}^{\left(b\right)}(x\prime ,x\prime \prime )$ be the estimated indirect effect when changing $x_{i}$ from x′ to x″ in bootstrap sample b=1,…,B. Then, a (1−α)100% confidence interval for ${\text{IE}}_{X_{i}}(x\prime ,x\prime \prime )$ has endpoints at the α/2 and 1−α/2 quantiles of the ${\hat{\text{IE}}}_{X_{i}}^{\left(b\right)}(x\prime ,x\prime \prime )$ distribution. For testing the hypothesis

(7) $$H_{0}:{\text{IE}}_{X_{i}}(x\prime ,x\prime \prime )=\Delta ,\;H_{A}:{\text{IE}}_{X_{i}}(x\prime ,x\prime \prime )\neq \Delta ,$$

let $p_{L}$ and $p_{U}$ be the proportion of bootstrap samples below and above Δ, respectively. Specifically, $p_{L}=B^{-1}\sum_{b=1}^{B}\;1_{{\hat{\text{IE}}}_{X_{i}}^{\left(b\right)}(x\prime ,x\prime \prime )<\Delta }$ and $p_{U}=B^{-1}\sum_{b=1}^{B}\;1_{{\hat{\text{IE}}}_{X_{i}}^{\left(b\right)}(x\prime ,x\prime \prime )>\Delta }$. Then, the *P*-value for hypothesis test (7) is $2\min(p_{L},p_{U})$. Similar procedures can be used to construct confidence intervals and test for direct effects. Following Efron and Tibshirani (1994, Chapter 13), we compute B=1000 bootstrap samples for making confidence intervals. Along the same line, we can make inference on partial correlation to gain insight into the mediator–mediator interaction (see Supplementary Section S2.2 for detail). Larger numbers of bootstrap samples could be used to ensure that the quantiles of the bootstrap samples better approximate the bootstrap sampling distribution, at the cost of additional computation time.

## 3 Simulations

### 3.1 Logistic models: bias

In the context of binary outcomes, we compare the performance of our method with two approximation methods that exploit the rare disease assumption (VanderWeele and Vansteelandt, 2010) and probit model (Gaynor *et al.*, 2018). With the logistic model with univariate mediator, if P(y=1)≈0 then ${\text{DE}}^{o}(x\prime ,x\prime +1)\approx \;\text{exp}(\alpha^{\left(X\right)})$ and ${\text{IE}}^{o}(x\prime ,x\prime +1)\approx \;\text{exp}(\beta^{\left(X\right)}\alpha^{\left(M\right)})$. One can then estimate these approximations via logistic regression estimates of $\alpha^{\left(X\right)}$ and $\beta^{\left(X\right)}$. This estimator is increasingly accurate as the disease becomes more rare, that is, P(y=1) converges to 0 (Huang *et al.*, 2014; VanderWeele and Vansteelandt, 2010). Gaynor *et al.* (2018) relax such assumption in the logistic outcome model by using a probit outcome model to approximate the logistic model, and then they also derive a closed-form expression of the direct and indirect effects. The confidence interval is calculated by a multivariate Delta method under the assumption of normality.

In the single mediator scenario, we compare the accuracy of these two methods with our approach using the logistic outcome model. We evaluate bias of direct and indirect effect estimators across various levels of disease prevalence, rare to common disease settings. Following Gaynor *et al.* (2018) (Section 3.1), we simulate

$$\begin{array}{c} c∼N(0.12,0.75^{2});\;\;x∼N(0.4,0.75^{2})\\ m|x,c∼N(0.1+0.5x+0.4c,0.75^{2})\\ \text{logit}(P(y=1|x,m,c))=k+0.4x+0.5m+0.25c. \end{array}$$

**Fig. 2.**
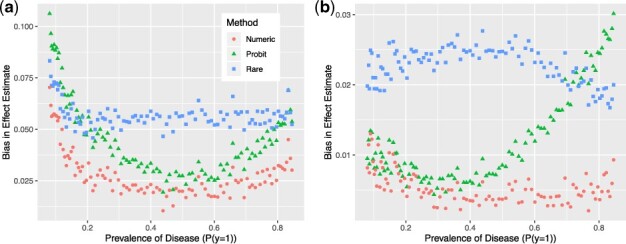
Comparison of methods for computing the (**a**) direct effect and (**b**) indirect effect with logistic models. Numeric approximation has lower bias than the rare disease approximation and the probit approximation

The constant k is varied to generate different prevalences p(y=1). At each level of k, we generate simulation data of sample size n=500 and evaluate bias of indirect and direct effects based on N=5000 replicate runs; the effect estimators are averaged across the N=5000 runs and the bias of the estimator is computed (Fig. 2).

Across all prevalence levels, our approach with numeric approximation consistently provides the lowest bias for both the direct and indirect effects. At low prevalence levels, the model with the rare disease assumption has lower bias than probit approximation for the indirect effect, but higher bias for the direct effect. For common diseases with prevalence around 0.5, the probit model performs much better than the model with the rare disease assumption, which is as expected.

### 3.2 Survival outcomes: Type 1 error and power

For survival outcomes, we evaluate type I error by varying the number of candidate mediators and sample sizes assuming that the true indirect effect is 0. For a binary exposure with a prevalence probability of 0.5, we assume that there are five mediators that are correlated with the exposure with $R^{2}=0.2$. We generate 0, 5, 15, 45, and 95 additional mediators independently from standard normal distributions that are uncorrelated with the exposure; the total numbers of candidate mediators of 5, 10, 20, 50, and 100 are considered in this simulation. The time-to-event response follows an exponential model with a Cox proportional hazards model coefficient of 0.5 for the exposure direct effect with 50% censoring, and all the candidate mediators have coefficients of 0. In this way, the hazard function depends only on the exposure but not on any of the mediators. This is because the true indirect effect is 0. We consider sample sizes of 50, 100, 200, 400 and the results are summarized based on 500 runs. We estimate the path coefficients using both regularized and non-regularized regressions, which are used for direct and indirect effects computations following Section 2.4. With our inferential procedure in Section 2.5, we evaluate the type I error by coverage probability, which is the proportion of replications that the estimated confidence interval covers the true indirect effect. In the null setting, 1− coverage probability is equivalent to a type I error. Supplementary Table S1 summarizes the coverage probabilities of estimators from both regularized and non-regularized regressions. Overall, both the methods control type I error with coverage probability near or above the nominal level.

We then evaluate the power of our method under various effect sizes. We keep the exposure–mediator and the exposure–outcome relationships the same as the type I error evaluation. For the mediator–outcome relationship, those five mediators correlated with the exposures are assigned non-zero coefficients of 0.2 and 0.1 for simulation settings with the strong and weak mediators in the power analysis, and the rest of the mediators are kept the same. Violin plots of the non-regularized results with strong mediators across various sample sizes are shown in Supplementary Figure S1. Empirically, the point estimates appear to be converging to the true indirect effect of −695 and −429 as samples sizes become larger for both strong and weak mediators’ scenarios, respectively.

**Fig. 3.**
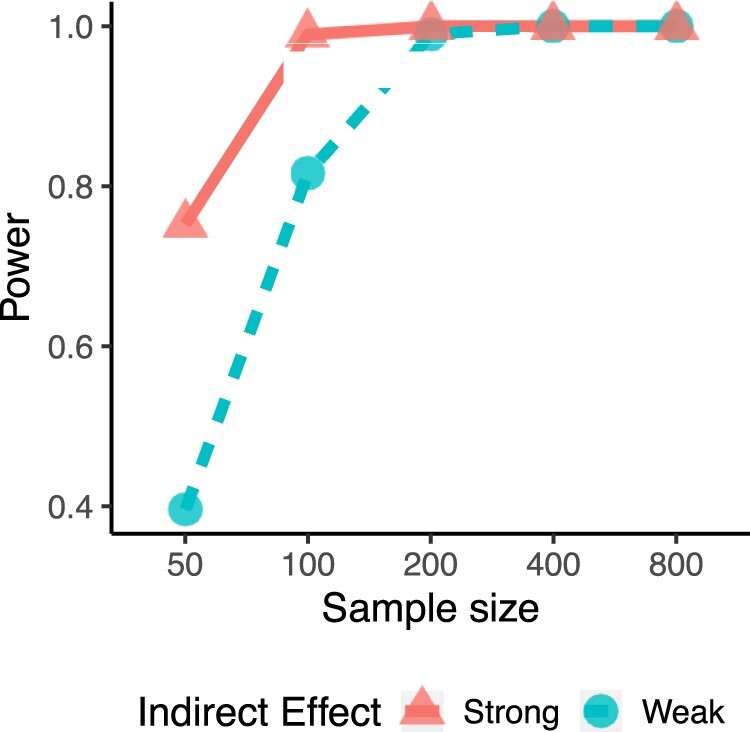
Power for simulation with strong mediators and weak mediators with ridge penalties for 100 mediators. The true indirect effect is −695 for the strong mediators and the true indirect effect is −429 for the weak mediators

With the inference procedure in Section 2.5, we evaluate coverage probabilities and power controlling a type I error of 0.05 (Supplementary Table S2). Most coverage probabilities are at the nominal level, except for those cases where the number of mediators are relatively large compared with the sample size. Power summarizes the proportion of replications for which that the confidence interval does not include 0, which measures the ability to correctly reject a null hypothesis that is indeed false. When sample sizes are less than 100, the non-regularized estimation provides low power across all numbers for mediators even when there are no noisy mediators. Power increases as the sample size gets larger. At sample size of 400, except for the case of weak mediators with 100 mediators, our approach without regularization achieves proper power. In addition, we evaluate the power using ridge penalties that can handle more number of mediators than the sample size (Supplementary Table S3 and Fig. 3). We observe favorable performance in terms of power using regularization—except for cases of weak mediators with sample size of 50 and number of mediators of 100, which are the most challenging simulation setup. The computational time in minutes is summarized in Supplementary Table S4 and detailed discussion is included in Supplementary Section S3.2. In a scenario similar to our data application, with sample size of 400 and number of mediators of 50, the computational times are around 1 and 12 minutes without and with using ridge regularization, respectively. In conclusion, although employing ridge estimation into our inference procedure requires more computational time, it helps to improve in detection of non-zero indirect effects in the presence of both weak and strong mediators.

## 4 Proteogenomic analyses in kidney cancer

TCGA studies have extensively investigated molecular changes in cancer patients at the genomic, epigenomic, transcriptomic and proteomic levels in relation to patients’ clinical data. KIRC is considered as the most common and lethal type of kidney cancer, and it has increasingly been identified as a metabolic disease and metabolic pathways are considered as therapeutic targets of intervention (Rathmell *et al.*, 2018). Among many other discoveries, TCGA Research Network (2013) identified key genes and pathways in the metabolic shifts in aggressive tumors, including TCA cycle, AMPK gene complex and the PTEN gene, the pentose phosphate pathway and PAS. (TCGA Research Network, 2013). We assess whether the causal effect of changes in these key genes within pathways at the mRNA level is mediated by changes at the protein expression level. Since increased mRNA expression levels have the ability to increase protein expression levels via translational mechanisms, it is sensible to view protein expression levels as potential causal mediators of the mRNA–survival relations. By exploiting the prior biologic knowledge of genes, we may have better chance to identify the genes and/or proteins involved in cancer progression (Wang *et al.*, 2010). In addition to mRNA–survival relations, we also evaluate mutation–survival relations (see Supplementary Section S4.1.2 for detail). A mutation has not only the ability to regulate the level of protein expressions but could also alter proteins’ function without altering the expression levels. Therefore, it is reasonable to view a mutation as a potential exposure and assess how much of its effect on survival is mediated by proteins.

We downloaded mRNA expression, somatic mutation and RPPA-based protein expression data using ‘DownloadRNASeqData’, ‘DownloadSomaticMutationData’ and ‘DownloadRPPAData’ functions, respectively, in the TCGA-assembler2 R package (Wei *et al.*, 2017). Pathways and the gene members involved in a metabolic shift for aggressive tumors from TCGA Research Network (2013) are summarized in Supplementary Table S5. The clinical endpoints include OS, DSS and PFI based on definitions in Liu *et al.* (2018) (Supplementary Fig. S2a–c). OS is defined as the period from the date of initial diagnosis until death from any cause. The censored time is from the date of initial diagnosis until the date of last contact. The DSS event time is from the date of initial diagnosis to the date of death from the disease and the censored time is from the date of initial diagnosis to the date of last contact or death from another cause. Noted that if a patient dies from a non-disease-related cause, then such individual is considered as right-censored sample. Hypothetically, a patient would experience a disease-related event no earlier than the death from any cause (see Supplementary Fig. S2a and b). PFI is defined as the period from the date of initial diagnosis until the date of the first occurrence of a new tumor event, which includes progression of the disease, locoregional recurrence, distant metastasis, new primary tumor or death with tumor. The event time is the shortest period from initial diagnosis to any of the events. The censored time is from the date of initial diagnosis to the date of last contact or the date of death without disease. Note that in this PFI definition, the events include death with tumor, but they do not include deaths from other causes, which is distinguished from the more often used endpoint progression-free survival (PFS) that does contain death from other causes as an event. The number of events related to these three outcomes is depicted in Supplementary Figure S2b. By definition, DSS events are at the intersection of those of OS and PFI, and DSS has the longest survival time among the three. Survival probabilities are estimated in Kaplan–Meier curves in Supplementary Figure S2c. DSS does not reach to its median survival time. The median survival time for OS and PFI is 2564 days ([2190, NA) 95% CI) and 3250 days ([2386, NA) 95% CI), respectively.

For each of the three endpoints, we aim to assess whether the causal effect on survival of changes in these key genes within pathways (Supplementary Table S5) related to the metabolic process at the mRNA level is mediated by changes at the protein expression level. Simulation studies (Section 3) suggest that for relatively small numbers of exposures and covariates compared with the sample sizes, our method produces reasonable parameter estimates and well-calibrated uncertainties. Using ridge penalty enables estimation of effects for high-dimensional mediators and attains higher power in detecting the presence of an indirect effect. We use restricted mean survival truncated to 2000 days given that the estimated median follow-up time is 1731 days (95% CI, 1525–1871) since restriction time up to median follow-up time is recommended in quality-adjusted survival analyses (Goldhirsch *et al.*, 1989; Martin and Simes, 2013).

**Fig. 4.**
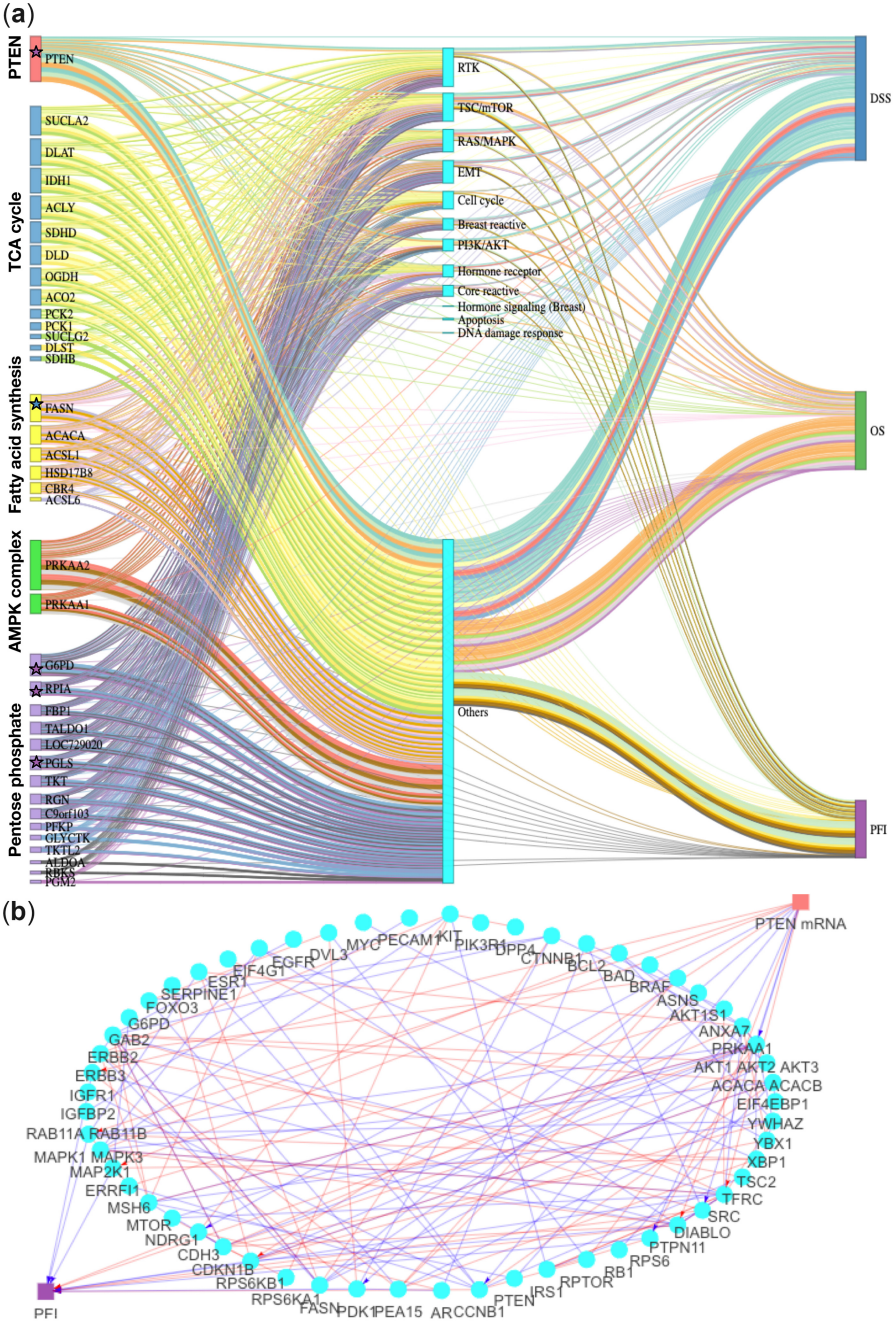
(**a**) Sankey diagram illustrates the indirect and direct effects (in days) of mRNA expression on three clinical survival outcomes as mediated by protein expressions (grouped into pathways). Nodes at the left are mRNA (colored coded by the pathways), cyan nodes at the middle are proteins (grouped into protein pathways), and nodes at the right are three survival endpoints. Edges are color coded by each of the mediation analyses with edge widths proportional to estimated absolute value of coefficients in regression without ridge penalties. Significant results in total/direct/indirect effect with ridge penalties are highlighted with a star that is in the color that indicates the corresponding survival outcome. (**b**) Multilayered network of PTEN gene on PFI mediated by proteins. A path PTEN → protein A → PFI is connected if protein A is a significant mediator and the magnitude of the product of the path coefficients is larger than 0.02. Within proteins, we connect two proteins if the *P*-value of its partial correlation is less than 0.001. Red indicates positive coefficients and blue indicates negative coefficients

For each of the survival outcomes and each of the pathways, we applied the following prescreening procedures: (1) we regressed the survival outcome on each of the genes within such pathway using the Cox model. Genes with marginal *P* < 0.01 were kept as candidate exposures. (2) Using the Cox model, we regressed the survival outcome on each of the proteins, adjusted for all the selected genes from step (1). Proteins with *P* < 0.01 were kept as candidate mediators. Supplementary Table S6 summarizes the number of exposures and mediators for each of the mediation analysis. The number of exposure(s) ranges from 1 to 12, and the number of mediators ranges from 15 to 72 across the total number of 15 (5 pathways × 3 outcomes) mediation analyses. The Sankey diagram (Fig. 4a) illustrates the information flow of a mediation process from each gene to each survival outcome mediated jointly by proteins that are sorted by key protein signaling pathways (Akbani *et al.*, 2014; Bhattacharyya *et al.*, 2020; Ha *et al.*, 2018). The total effect of mRNA on survival outcomes can be divided into two paths: one path from mRNA to proteins and then to survival outcome (indirect effect) and another path from mRNA to the survival outcome through other biological processes other than those mediators (direct effect). Edge widths are proportional to absolute values of path coefficients. The estimated effects and the 95% confidence intervals are in Supplementary Tables S7–S9. We select genes that have significant indirect effects using regularized regressions since it provides high power in detecting non-zero indirect effects (Section 3.2).

The direct effects of several genes have the same directions as found in TCGA Research Network (2013). For example, fatty acid synthase (FASN) in the fatty acid pathway has a significant direct effect on DSS at α=0.05. On average, while holding other variables constant, one unit increase in the expression value in FASN is associated with 52 days less DSS time without being mediated by those proteins during 2000 days of follow-up (Supplementary Table S9). In addition, 6-phosphogluconolactonase (PGLS) in the pentose phosphate pathway has a total effect of reducing the mean-restricted lifetime of PFI by 129 days, and the effect appears to be primarily direct (Supplementary Table S8).

Besides, PTEN has a significant indirect effect on PFI; however, its direct effect is not significant (Supplementary Table S7). We further identify mediation effects of proteins. From the Sankey diagram (Fig. 4a), proteins in receptor tyrosine kinase (RTK) and phosphatidylinositol 3‒kinase (PI3K)/protein kinase B (AKT) mediate PTEN’s effect on PFI to a large extent. On the other hand, the effect of PTEN on PFI does not pass through proteins in DNA damage response. At α=0.05, significant mediators of the effect of PTEN on PFI include several proteins in PI3K/AKT, tuberous sclerosis complex (TSC)/mammalian target of rapamycin (mTOR), RAS/MAPK, and RTK protein pathways (Supplementary Table S10). This result is consistent with known biological mechanism that the tumor suppressor PTEN is an upstream regulator of PI3K/AKT (Carracedo and Pandolfi, 2008; Chu and Tarnawski, 2004; Kanehisa and Goto, 2000). Figure 4b presents the multilayer casual network for PTEN gene on PFI mediated by translational mechanisms, and within-mediator interactions. We find that proteins in PI3K/AKT pathways are significantly correlated with several proteins in RAS/MAPK and TSC/mTOR pathways (Fig. 4b), which are known to be co-expressed: the cross-talk between PI3K/AKT–TSC/mTOR signaling pathways is a critical biological process regulating cell survival, proliferation and motility (Carracedo and Pandolfi, 2008; Kanehisa and Goto, 2000); and PI3K/AKT and RAS/MAPK signaling pathways tightly regulate mammalian target of rapamycin complex 1 (mTORC1) and RTK (Carracedo *et al.*, 2008; Kurtzeborn *et al.*, 2019; Mendoza *et al.*, 2011).

Supplementary Table S11 and Supplementary Figure S4 summarize the indirect, direct and total effects and 95% confidence intervals (in days) of a mutation as mediated by protein expression for PFI in KIRC. BRCA1-associated protein 1 (BAP1) mutation has a significant total effect on OS, PFI and DSS, which has the same direction as reported in TCGA Research Network (2013). It is associated with worse survival outcomes (Supplementary Table S11). BAP1 also has a significant indirect effect on OS and DSS. Proteins that significantly mediate the mutation’s effect on survival are summarized in Supplementary Table S12. On average, BAP1 mutation has a total effect of reducing mean-restricted lifetime in OS by 300 days, with 234 days explained by changes in protein mediators, 78% (234/300) of the total effect. Besides, it has a total effect of reducing mean-restricted lifetime of DSS by 374 days, with 227 days explained by changes in protein mediators, 61% (227/374) of the total effect. Although BAP1 has been reported to be a predictive biomarker of tumor progression in several cancer types, its clinical role remains unknown (Gulati *et al.*, 2022; Kuznetsov *et al.*, 2019). Harbour *et al.* (2010) report that the loss of BAP1 is associated with the mRNA expression level of catenin beta 1 (CTNNB1), which plays a critical role in cell cycles (Li *et al.*, 2009). It is reasonable that CTNNB1 is a significant mediator of the BAP1 mutation on survival (Supplementary Table S11 and Supplementary Fig. S4b and e). Regarding mTOR mutation as exposure, its indirect effect significantly reduces mean-restricted lifetime of OS by 154 days (Supplementary Table S11). mTOR mutations are associated with increased mTORC1/2 pathway activity (Grabiner *et al.*, 2014). We observe that MAPK1, MAPK3, ribosomal protein S6 kinase A1 (RPS6KA1) and Y-box binding protein 1 (YBX1) in the pathway RAS/MAPK significantly mediate the effect of mTOR on OS, and those proteins are also correlated with each other (Supplementary Table S12 and Supplementary Figure S4c). This result is expected since mTORC1 is upregulated by RAS/MAPK signaling pathways (Carracedo *et al.*, 2008; Kurtzeborn *et al.*, 2019; Mendoza *et al.*, 2011).

## 5 Discussion

We proposed a general methodology of mediation analysis for data observed from random variables that form a multilayered structure where the layers are naturally ordered and unknown correlation structures are present within layer. Our method can handle different choices of outcomes such as continuous, binary and survival, and measured on the mean, odds and restricted mean scales from linear, logistic and Cox-proportional hazards models, respectively. The proposed framework has advantages over existing approaches such as not requiring assumptions on disease prevalence (rare or common disease assumptions) in the case of binary outcomes and accommodating continuous exposure variables (x), such as mRNA expression. The framework controls for confounders and accommodates correlated mediators without requiring assumptions on the direction of any mediator causal structure. Our mediateR package makes these models easily accessible to users.

Bootstrap-based inferential procedure can be used to assess the existence and likely ranges for the direct and indirect effects that are evaluated from (non-)regularized regression models. Simulation studies suggest that, for relatively small numbers of mediators compared with the sample sizes, the models with non-regularized regression produce reasonable parameter estimates and well-calibrated uncertainties. For high-dimensional mediators, shrinking the estimated parameters toward 0 using the ridge penalty attains higher power in detecting the presence of an indirect effect. In choosing the ridge penalty, we hypothesize that there are many causal variants, and each have a small contribution to the response (Boyle *et al.*, 2017; Goldstein, 2009). The ridge penalty provides similar coefficients for highly correlated predictors, rather than selecting a few representative ones among a set of highly correlated predictors (Friedman *et al.*, 2010).

The causal interpretation of direct and indirect effects requires strong causal assumptions in Supplementary Section S1, including no unmeasured confounders. The RPPA platform quantifies protein expression based on antibodies that target nearly 200 predefined proteins (Li *et al.*, 2013). If an unmeasured true protein mediates the effect of exposure to the outcome, the effect of the indirect path mRNA → protein A → response is added to the direct effect, which is interpreted as the effect of unspecified causal mechanisms. Thus, the mass spectrometry (MS)-based data that cover a wider spectrum of protein quantifications will help to identify more insightful mediating mechanisms. Besides, technical challenges may affect the quality of RPPA, including (1) the quality of reference antibodies and (2) the spatial heterogeneity of sampled tumor regions (Boellner and Becker, 2015). The presence of measurement error may weaken the mediator–outcome effect and then lead to an underestimated indirect effect. This may be a possible explanation for the cases where an mRNA exhibits a significant direct effect even though the gene’s protein product is considered as a mediator. To a limited extent, these causal assumptions in Supplementary Section S1 can be checked, and violations addressed, with additional modeling. For example, sensitivity analyses can be used to test for unobserved pre-exposure covariates (Imai *et al.*, 2010b) and a mediator measurement error which biases effect size estimates and can be corrected via regression calibration (Valeri *et al.*, 2014).

## Supplementary Material

btad023_Supplementary_Data

## Data Availability

The data underlying this article are available in National Cancer Institute Genomic Data Commons Data Portal at https://portal.gdc.cancer.gov/.

## References

[btad023-B1] Akbani R. et al (2014) A pan-cancer proteomic perspective on the cancer genome atlas. Nat. Commun., 5, 1–15. 10.1038/ncomms4887 PMC4109726 24871328

[btad023-B2] Alcaraz N. et al (2017) De novo pathway-based biomarker identification. Nucleic Acids Res., 45, e151. 28934488 10.1093/nar/gkx642 PMC5766193

[btad023-B3] Avin C. et al (2005) Identifiability of path-specific effects. In Proceedings of the 19th International Joint Conference on Artificial Intelligence. Morgan Kaufmann Publishers Inc., San Francisco, CA, USA. pp. 357–363.

[btad023-B4] Barfield R. et al (2017) Testing for the indirect effect under the null for genome-wide mediation analyses. Genet. Epidemiol., 41, 824–833. 29082545 10.1002/gepi.22084 PMC5696067

[btad023-B5] Baron R.M. , Kenny D.A. (1986) The moderator–mediator variable distinction in social psychological research: Conceptual, strategic, and statistical considerations. J. Pers. Soc. Psychol., 51, 1173–1182. 3806354 10.1037//0022-3514.51.6.1173

[btad023-B6] Bhattacharyya R. et al (2020) Personalized network modeling of the pan-cancer patient and cell line interactome. JCO Clin. Cancer Inform., 4, 399–411. 32374631 10.1200/CCI.19.00140 PMC7265783

[btad023-B7] Boehm J.S. , Hahn W.C. (2011) Towards systematic functional characterization of cancer genomes. Nat. Rev. Genet., 12, 487–498. 21681210 10.1038/nrg3013

[btad023-B8] Boellner S. , Becker K.-F. (2015) Reverse phase protein arrays—quantitative assessment of multiple biomarkers in biopsies for clinical use. Microarrays, 4, 98–114. 27600215 10.3390/microarrays4020098 PMC4996393

[btad023-B9] Boyle E.A. et al (2017) An expanded view of complex traits: From polygenic to omnigenic. Cell, 169, 1177–1186. 28622505 10.1016/j.cell.2017.05.038 PMC5536862

[btad023-B10] Carracedo A. , Pandolfi P. (2008) The PTEN–PI3K pathway: Of feedbacks and cross-talks. Oncogene, 27, 5527–5541. 18794886 10.1038/onc.2008.247

[btad023-B11] Carracedo A. et al (2008) Inhibition of mTORC1 leads to MAPK pathway activation through a PI3K-dependent feedback loop in human cancer. J. Clin. Invest., 118, 3065–3074. 18725988 10.1172/JCI34739 PMC2518073

[btad023-B12] Chen P.-Y. , Tsiatis A.A. (2001) Causal inference on the difference of the restricted mean lifetime between two groups. Biometrics, 57, 1030–1038. 11764241 10.1111/j.0006-341x.2001.01030.x

[btad023-B13] Chu E.C. , Tarnawski A.S. (2004) PTEN regulatory functions in tumor suppression and cell biology. Med. Sci. Monit., 10, RA235–41. 15448614

[btad023-B14] Efron B. , Tibshirani R.J. (1994) An Introduction to the Bootstrap. CRC Press.

[btad023-B15] Fasanelli F. et al (2019) Marginal time-dependent causal effects in mediation analysis with survival data. Am. J. Epidemiol., 188, 967–974. 30689682 10.1093/aje/kwz016

[btad023-B16] Friedman J.H. et al (2010) Regularization paths for generalized linear models via coordinate descent. J. Stat. Softw., 33, 1–22. 20808728 PMC2929880

[btad023-B17] Gaynor S.M. et al (2018) Mediation analysis for common binary outcomes. Stat. Med., **38**(4), 512–529. 30256434 10.1002/sim.7945

[btad023-B18] Goldhirsch A. et al (1989) Costs and benefits of adjuvant therapy in breast cancer: A quality-adjusted survival analysis. J. Clin. Oncol., 7, 36–44. 2642538 10.1200/JCO.1989.7.1.36

[btad023-B19] Goldstein D.B. (2009) Common genetic variation and human traits. N. Engl. J. Med., 360, 1696–1698. 19369660 10.1056/NEJMp0806284

[btad023-B20] Grabiner B.C. et al (2014) A diverse array of cancer-associated MTOR mutations are hyperactivating and can predict rapamycin sensitivity cancer-associated hyperactivating MTOR mutations. Cancer Discov., 4, 554–563. 24631838 10.1158/2159-8290.CD-13-0929 PMC4012430

[btad023-B21] Gulati S. et al (2022) BRCA1-associated protein 1 (BAP-1) as a prognostic and predictive biomarker in clear cell renal cell carcinoma: A systematic review. Kidney Cancer (Preprint), 1–13.

[btad023-B22] Ha M.J. et al (2018) Personalized integrated network modeling of the cancer proteome atlas. Sci. Rep., 8, 1–14. 30297783 10.1038/s41598-018-32682-x PMC6175854

[btad023-B23] Harbour J.W. et al (2010) Frequent mutation of BAP1 in metastasizing uveal melanomas. Science, 330, 1410–1413. 21051595 10.1126/science.1194472 PMC3087380

[btad023-B24] Huang Y.-T. , Pan W.-C. (2016) Hypothesis test of mediation effect in causal mediation model with high-dimensional continuous mediators. Biometrics, 72, 402–413. 26414245 10.1111/biom.12421

[btad023-B25] Huang Y.-T. et al (2014) Joint analysis of SNP and gene expression data in genetic association studies of complex diseases. Ann. Appl. Stat., 8, 352–376. 24729824 10.1214/13-AOAS690 PMC3981558

[btad023-B26] Imai K. et al (2010a) A general approach to causal mediation analysis. Psychol. Methods, 15, 309–334. 20954780 10.1037/a0020761

[btad023-B27] Imai K. et al (2010b) Identification, inference and sensitivity analysis for causal mediation effects. Stat. Sci., **25**(1), 51–71.

[btad023-B28] Kanehisa M. , Goto S. (2000) Kegg: Kyoto encyclopedia of genes and genomes. Nucleic Acids Res., 28, 27–30. 10592173 10.1093/nar/28.1.27 PMC102409

[btad023-B29] Kumar D. et al (2016) Integrating transcriptome and proteome profiling: Strategies and applications. Proteomics, 16, 2533–2544. 27343053 10.1002/pmic.201600140

[btad023-B30] Kurtzeborn K. et al (2019) MAPK/ERK signaling in regulation of renal differentiation. Int. J. Mol. Sci., 20, 1779. 30974877 10.3390/ijms20071779 PMC6479953

[btad023-B31] Kuznetsov J.N. et al (2019) BAP1 regulates epigenetic switch from pluripotency to differentiation in developmental lineages giving rise to BAP1-mutant cancers. Sci. Adv., 5, eaax1738. 31555735 10.1126/sciadv.aax1738 PMC6750916

[btad023-B32] Li H. et al (2009) Down-regulation of death-associated protein kinase-2 is required for *β*-catenin-induced anoikis resistance of malignant epithelial cells. J. Biol. Chem., 284, 2012–2022. 18957423 10.1074/jbc.M805612200

[btad023-B33] Li J. et al (2013) TCPA: A resource for cancer functional proteomics data. Nat. Methods, 10, 1046–1047. 10.1038/nmeth.2650 PMC4076789 24037243

[btad023-B34] Liu J. et al; Cancer Genome Atlas Research Network. (2018) An integrated TCGA pan-cancer clinical data resource to drive high-quality survival outcome analytics. Cell, 173, 400–416.e11. 29625055 10.1016/j.cell.2018.02.052 PMC6066282

[btad023-B35] Martin A.J. , Simes R.J. (2013) Quality-adjusted survival as an end point in breast cancer trials. Clin. Invest., 3, 545–555.

[btad023-B36] Mendoza M.C. et al (2011) The RAS–ERK and PI3K–MTOR pathways: Cross-talk and compensation. Trends Biochem. Sci., 36, 320–328. 21531565 10.1016/j.tibs.2011.03.006 PMC3112285

[btad023-B37] Nesvizhskii A.I. (2014) Proteogenomics: Concepts, applications and computational strategies. Nat. Methods, 11, 1114–1125. 25357241 10.1038/nmeth.3144 PMC4392723

[btad023-B38] Pearl J. (2001). Direct and indirect effects. In Proceedings of the Seventeenth Conference on Uncertainty in Artificial Intelligence. Morgan Kaufmann Publishers Inc., pp. 411–420.

[btad023-B39] Pearl J. (2009). Causality. Cambridge University Press.

[btad023-B40] Rathmell W.K. et al (2018) Metabolic pathways in kidney cancer: Current therapies and future directions. J. Clin. Oncol., 36, 3540–3546. 10.1200/JCO.2018.79.2309 PMC6488445 30372395

[btad023-B41] Rijnhart J.J. et al (2021) Mediation analysis methods used in observational research: A scoping review and recommendations. BMC Med. Res. Methodol., 21, 1–17. 34689754 10.1186/s12874-021-01426-3 PMC8543973

[btad023-B42] Robins J.M. , Greenland S. (1992) Identifiability and exchangeability for direct and indirect effects. Epidemiology, 3, 143–155. 1576220 10.1097/00001648-199203000-00013

[btad023-B43] Rodriguez H. et al (2021) The next horizon in precision oncology: Proteogenomics to inform cancer diagnosis and treatment. Cell, 184, 1661–1670. 33798439 10.1016/j.cell.2021.02.055 PMC8459793

[btad023-B44] Simon N. et al (2011) Regularization paths for Cox’s proportional hazards model via coordinate descent. J. Stat. Softw., 39, 1–13. 10.18637/jss.v039.i05 PMC4824408 27065756

[btad023-B45] Szklarczyk D. et al (2021) The string database in 2021: Customizable protein–protein networks, and functional characterization of user-uploaded gene/measurement sets. Nucleic Acids Res., 49, D605–D612. 33237311 10.1093/nar/gkaa1074 PMC7779004

[btad023-B46] Tang Z. et al (2017) GEPIA: A web server for cancer and normal gene expression profiling and interactive analyses. Nucleic Acids Res., 45, W98–W102. 28407145 10.1093/nar/gkx247 PMC5570223

[btad023-B47] TCGA Research Network. (2013) Comprehensive molecular characterization of clear cell renal cell carcinoma. Nature, 499, 43–49. 23792563 10.1038/nature12222 PMC3771322

[btad023-B48] Tchetgen E.J.T. , Shpitser I. (2012) Semiparametric theory for causal mediation analysis: Efficiency bounds, multiple robustness, and sensitivity analysis. Ann. Stat., 40, 1816–1845. 26770002 10.1214/12-AOS990 PMC4710381

[btad023-B49] Tein J.-Y. , MacKinnon D.P. (2003) Estimating mediated effects with survival data. In: Yanai,H. *et al*. (eds), New Developments in Psychometrics, Springer, Tokyo, Japan, pp. 405–412.

[btad023-B50] Uno H. et al (2014) Moving beyond the hazard ratio in quantifying the between-group difference in survival analysis. J. Clin. Oncol., 32, 2380–2385. 24982461 10.1200/JCO.2014.55.2208 PMC4105489

[btad023-B51] Valeri L. et al (2014) Mediation analysis when a continuous mediator is measured with error and the outcome follows a generalized linear model. Stat. Med., 33, 4875–4890. 25220625 10.1002/sim.6295 PMC4224977

[btad023-B52] VanderWeele T.J. (2011) Causal mediation analysis with survival data. Epidemiology (Cambridge, MA), 22, 582–585. 10.1097/EDE.0b013e31821db37e PMC3109321 21642779

[btad023-B53] VanderWeele T.J. , Vansteelandt S. (2010) Odds ratios for mediation analysis for a dichotomous outcome. Am. J. Epidemiol., 172, 1339–1348. 21036955 10.1093/aje/kwq332 PMC2998205

[btad023-B54] VanderWeele T.J. et al (2014) Effect decomposition in the presence of an exposure-induced mediator-outcome confounder. Epidemiology (Cambridge, MA), 25, 300–306. 10.1097/EDE.0000000000000034 PMC4214081 24487213

[btad023-B55] Wang K. et al (2010) Analysing biological pathways in genome-wide association studies. Nat. Rev. Genet., 11, 843–854. 21085203 10.1038/nrg2884

[btad023-B56] Wei L. et al (2017) TCGA-assembler 2: Software pipeline for retrieval and processing of TCGA/CPTAC data. Bioinformatics, 34, 1615–1617. 10.1093/bioinformatics/btx812 PMC5925773 29272348

[btad023-B57] Yuan Y. et al (2014) Assessing the clinical utility of cancer genomic and proteomic data across tumor types. Nat. Biotechnol., 32, 644–652. 24952901 10.1038/nbt.2940 PMC4102885

[btad023-B58] Zhang H. et al (2021) Mediation analysis for survival data with high-dimensional mediators. Bioinformatics, 37, 3815–3821. 34343267 10.1093/bioinformatics/btab564 PMC8570823

[btad023-B59] Zhao Y. et al (2020) Sparse principal component based high-dimensional mediation analysis. Comput. Stat. Data Anal., 142, 106835. 32863492 10.1016/j.csda.2019.106835 PMC7449232

