# Supplementary material for: A unified mediation analysis framework for integrative cancer proteogenomics with clinical outcomes
Source: Bioinformatics. 2023 Jan 17;39(1):btad023. doi: 10.1093/bioinformatics/btad023 (PMC9879726; doi:10.1093/bioinformatics/btad023)
Supplement: btad023_Supplementary_Data [file btad023_supplementary_data.pdf]

Supplementary Materials for "A Unified Mediation Analysis Framework for Integrative Cancer Proteogenomics with Clinical Outcomes"

1 Counterfactual Random Variables and Assumptions

The following counterfactual independence and consistency relations are needed to express direct and indirect effects in terms of the joint distribution. For X_i for $i = 1, \dots, p$:

(1) $\boldsymbol{M}^{X_i=x'} \perp\!\!\!\perp X_i | \boldsymbol{C}, \boldsymbol{X}_{-i} \ \forall \ x'$: The value $\boldsymbol{M}$ obtains when X_i is set to x' is independent of X_i given $\boldsymbol{C}$ and all other exposures $\boldsymbol{X}_{-i}$.

(2) $Y^{X_i=x'',\boldsymbol{m}'} \perp\!\!\!\perp \boldsymbol{M}^{X_i=x'} | \boldsymbol{C}, \boldsymbol{X}_{-i} \ \forall \ x', x'', \boldsymbol{m}'$: The value that Y obtains when X_i is set to x'' and $\boldsymbol{M}$ is set to $\boldsymbol{m}'$ is independent of the value of $\boldsymbol{M}$ when it is set to the value it would take had X_i been x', given $\boldsymbol{C}$ and other exposures $\boldsymbol{X}_{-i}$.

(3) $Y^{X_i=x'',\boldsymbol{m}'} \perp\!\!\!\perp X_i | \boldsymbol{C}, \boldsymbol{X}_{-i} \ \forall \ x'', \boldsymbol{m}'$: The value that Y obtains when X_i is set to x'' and $\boldsymbol{M}$ is set to $\boldsymbol{m}'$ is independent of X_i, given $\boldsymbol{C}$ and other exposures $\boldsymbol{X}_{-i}$.

(4) $Y^{X_i=x'',\boldsymbol{m}'} \perp\!\!\!\perp \boldsymbol{M} | \boldsymbol{C}, \boldsymbol{X} \ \forall \ x'', \boldsymbol{m}'$: The value that Y obtains when X_i is set to x'' and $\boldsymbol{M}$ is set to $\boldsymbol{m}'$ is independent of $\boldsymbol{M}$, given $\boldsymbol{C}$ and other exposures $\boldsymbol{X}$.

(5) $X_i = x' \implies \boldsymbol{M}^{X_i=x'} = \boldsymbol{M} \ \forall \ x'$ (consistency): If $X_i = x'$ then the value that $\boldsymbol{M}$ takes when setting $X_i = x'$ is $\boldsymbol{M}$.

(6) $\boldsymbol{M} = \boldsymbol{m}', X_i = x' \implies Y^{X_i=x',\boldsymbol{m}'} = Y$ (consistency): If $X_i = x'$ and $\boldsymbol{M} = \boldsymbol{m}'$, then the value that $\boldsymbol{Y}$ takes when setting $X_i = x'$ is $\boldsymbol{M} = \boldsymbol{m}'$ is equal to Y.

(7) $Y^{X_i=x'} = Y^{X_i=x',\boldsymbol{m}^{X_i=x'}} \ \forall \ x'$ (composition)

These relations can be viewed as assumptions regarding the elemental counterfactual random variables, following the potential outcomes framework of Rubin (1974), or as consequences of the assumptions encoded in the causal DAG structure, following the approach of Pearl *et al.* (2009) (Section 7.3 p. 228). We refer to VanderWeele and Vansteelandt (2009) for additional discussion of these assumptions.

2 Causal Mediation Analysis Framework

2.1 Proof of direct effect

Let $g : \mathbb{R}^1 \to \mathbb{R}^1$. In mean difference scale, $g(\cdot) = \cdot$, and in restricted mean difference scale, $g(\cdot) = \min(\cdot, L)$. We have

$$\mathbb{E}[g(Y^{X_i=x'',\boldsymbol{M}^{X_i=x'}})] = \int_y g(y) p(Y^{X_i=x'',\boldsymbol{M}^{X_i=x'}} = y)$$

$$= \int_{y,\boldsymbol{x}_{-i},\boldsymbol{m},\boldsymbol{c}} g(y) \underbrace{p(Y^{X_i=x'',\boldsymbol{M}^{X_i=x'}} = y | \boldsymbol{x}_{-i}, \boldsymbol{M}^{X_i=x'} = \boldsymbol{m}, \boldsymbol{c})}_{\equiv A} \underbrace{p(\boldsymbol{x}_{-i}, \boldsymbol{M}^{X_i=x'} = \boldsymbol{m}, \boldsymbol{c})}_{\equiv B}. \quad (1)$$

Numbers above equals signs reference the counterfactual assumption used in the paper. We have

$$\begin{aligned}
B &= p(\boldsymbol{M}^{x_i=x'} = \boldsymbol{m}|\boldsymbol{x}_{-i}, \boldsymbol{c})p(\boldsymbol{x}_{-i}, \boldsymbol{c}) \\
&\overset{1}{=} p(\boldsymbol{M}^{x_i=x'} = \boldsymbol{m}|X_i = x', \boldsymbol{x}_{-i}, \boldsymbol{c})p(\boldsymbol{x}_{-i}, \boldsymbol{c}) \\
&\overset{5}{=} p(\boldsymbol{M} = \boldsymbol{m}|X_i = x', \boldsymbol{x}_{-i}, \boldsymbol{c})p(\boldsymbol{x}_{-i}, \boldsymbol{c}) \\
&= p(\boldsymbol{m}|x', \boldsymbol{x}_{-i}, \boldsymbol{c})p(\boldsymbol{x}_{-i}, \boldsymbol{c}).
\end{aligned}$$

Next we have

$$\begin{aligned}
A &= p(Y^{X_i=x'',\boldsymbol{m}} = y|\boldsymbol{x}_{-i}, \boldsymbol{M}^{X_i=x'} = \boldsymbol{m}, \boldsymbol{c}) \\
&\overset{2}{=} p(Y^{X_i=x'',\boldsymbol{m}} = y|\boldsymbol{x}_{-i}, \boldsymbol{c}) \\
&\overset{3}{=} p(Y^{X_i=x'',\boldsymbol{m}} = y|X_i = x'', \boldsymbol{x}_{-i}, \boldsymbol{c}) \\
&\overset{4}{=} p(Y^{X_i=x'',\boldsymbol{m}} = y|X_i = x'', \boldsymbol{x}_{-i}, \boldsymbol{m}, \boldsymbol{c}) \\
&\overset{6}{=} p(Y = y|X_i = x'', \boldsymbol{x}_{-i}, \boldsymbol{m}, \boldsymbol{c}) \\
&= p(y|x'', \boldsymbol{x}_{-i}, \boldsymbol{m}, \boldsymbol{c}).
\end{aligned}$$

Plugging the derivations for A and B back into Expression (1) we have

$$\begin{aligned}
&= \int_{y,\boldsymbol{x}_{-i},\boldsymbol{m},\boldsymbol{c}} g(y)p(y|x'', \boldsymbol{x}_{-i}, \boldsymbol{m}, \boldsymbol{c})p(\boldsymbol{m}|x', \boldsymbol{x}_{-i}, \boldsymbol{c})p(\boldsymbol{x}_{-i}, \boldsymbol{c}) \\
&= \int_{\boldsymbol{x}_{-i},\boldsymbol{m},\boldsymbol{c}} \mathbb{E}[g(Y)|x'', \boldsymbol{x}_{-i}, \boldsymbol{m}, \boldsymbol{c}]p(\boldsymbol{m}|x', \boldsymbol{x}_{-i}, \boldsymbol{c})p(\boldsymbol{x}_{-i}, \boldsymbol{c}).
\end{aligned}$$

We have

$$\mathbb{E}[g(Y^{X_i=x'})] \overset{7}{=} \mathbb{E}[g(Y^{X_i=x',\boldsymbol{M}^{X_i=x'}})] = \int_{\boldsymbol{x}_{-i},\boldsymbol{m},\boldsymbol{c}} \mathbb{E}[g(Y)|x', \boldsymbol{x}_{-i}, \boldsymbol{m}, \boldsymbol{c}]\underbrace{p(\boldsymbol{m}|x', \boldsymbol{x}_{-i}, \boldsymbol{c})p(\boldsymbol{x}_{-i}, \boldsymbol{c})}_{\equiv D}.$$

2.2 Within-layer interaction

The $\boldsymbol{\Sigma}_\epsilon$ in the mediator model (3) in section 2.3 is partial covariance after adjusting for the effects of covariates and exposure. We standardize it to a partial correlation matrix and denote it as $\boldsymbol{\Sigma_0}$. Its inverse, $\boldsymbol{\Sigma_0^{-1}}$ is the partial correlation after adjusting for covariates, exposures, and all other mediators. We can use a similar procedure described in section 2.5 to conduct hypothesis testing. Let γ_{lk} denote lth row and kth column of the partial correlation matrix, which measures the interaction between M_l and M_k. For each of the bootstrap samples $b = \{1, \ldots, B\}$, we compute the estimated partial correlation $\hat{\gamma}_{lk}^{(b)}$. To test the null hypothesis $H_0 : \gamma_{lk} = 0$, let p_L and p_U be the proportion of bootstrap samples below and above Δ, respectively. Specifically $p_L = B^{-1}\sum_{b=1}^{B} \mathbb{1}_{\hat{\gamma}_{lk}^{(b)}<0}$ and $p_U = B^{-1}\sum_{b=1}^{B} \mathbb{1}_{\hat{\gamma}_{lk}^{(b)}>0}$. Then the p-value for hypothesis test is $2\min(p_L, p_U)$. Following Efron and Tibshirani (1994) Chapter 13, we compute $B = 1000$ bootstrap samples for making confidence intervals.

3 Simulations

3.1 Survival outcome

		Coverage Probabilities	
n	No. Mediators	Cox regression	Ridge regression
50	5	0.97	1.00
50	10	0.98	1.00
50	20	1.00	1.00
50	50	-	1.00
50	100	-	1.00
100	5	0.97	1.00
100	10	0.97	1.00
100	20	0.99	1.00
100	50	1.00	1.00
100	100	-	1.00
200	5	0.96	1.00
200	10	0.95	1.00
200	20	0.97	1.00
200	50	1.00	1.00
200	100	1.00	0.99
400	5	0.94	1.00
400	10	0.95	1.00
400	20	0.96	1.00
400	50	0.97	1.00
400	100	1.00	0.98
800	5	0.94	1.00
800	10	0.94	1.00
800	20	0.96	1.00
800	50	0.97	1.00
800	100	0.97	0.99

Table S1: Empirical coverage probabilities for 95% confidence intervals in the null simulation (indirect effect=0).

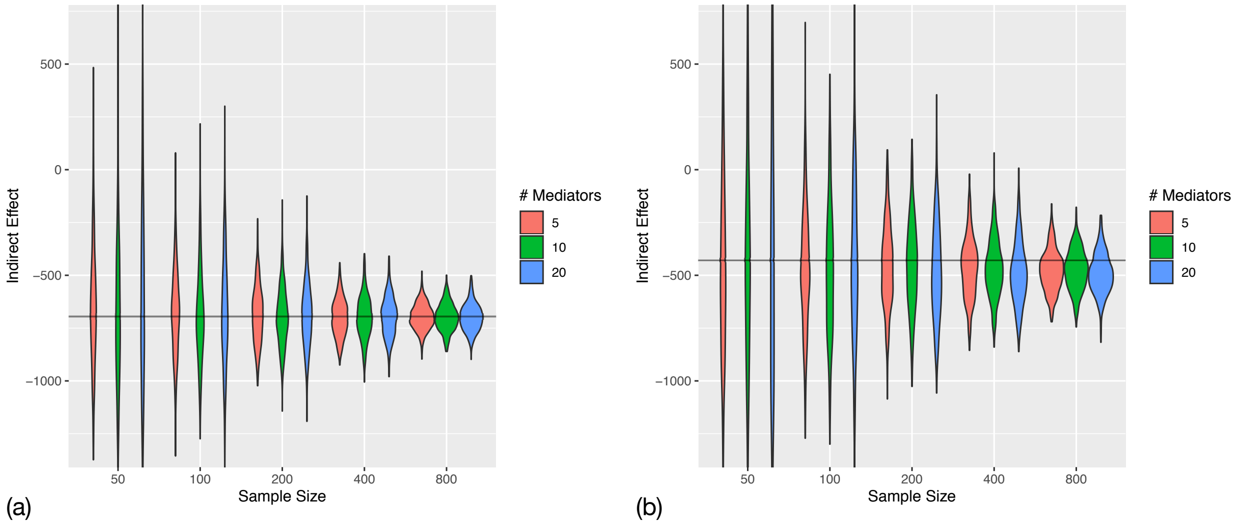

Figure S1: (a) Indirect effect point estimates by non-regularized regression for 500 runs with large indirect effect. Horizontal line is true IE. (b) Indirect effect point estimates by non-regularized regression for 500 runs with small indirect effect. Horizontal line is true IE.

n	No. Med.	Weak Mediators Cov	Weak Mediators Power	Strong Mediators Cov	Strong Mediators Power
50	5	0.98	0.13	0.99	0.34
50	10	0.99	0.06	0.99	0.15
50	20	1.00	0.00	0.98	0.00
100	5	0.96	0.31	0.97	0.79
100	10	0.97	0.23	0.97	0.68
100	20	0.99	0.10	0.99	0.41
100	50	1.00	0.00	0.99	0.00
200	5	0.95	0.56	0.96	0.98
200	10	0.95	0.47	0.97	0.98
200	20	0.96	0.42	0.98	0.93
200	50	1.00	0.14	1.00	0.64
200	100	1.00	0.00	1.00	0.00
400	5	0.95	0.87	0.98	1.00
400	10	0.94	0.83	0.94	1.00
400	20	0.94	0.82	0.98	1.00
400	50	0.97	0.67	0.98	1.00
400	100	1.00	0.34	0.99	0.96
800	5	0.92	0.99	0.97	1.00
800	10	0.93	0.99	0.96	1.00
800	20	0.94	0.99	0.97	1.00
800	50	0.94	0.98	0.97	1.00
800	100	0.97	0.95	0.98	1.00

Table S2: Empirical coverage probabilities and power for simulation with strong mediators and weak mediators without regularization. The true indirect effect is -695 for the strong mediators, and the true indirect effect is -429 for the weak mediators.

3.2 Computational time

The computation time and complexity of our algorithm depend on ridge regressions to handle high-dimensional mediators. For tuning parameters in both mediator and outcome models with ridge penalties, we adopt the 10-fold cross-validation with grid search of 100 candidates between the λ_{min} and λ_{max} in *glmnet* R package, where λ_{min} gives the model closed to unpenalized regression and λ_{max} penalize all the coefficients to 0 ((Simon *et al.*, 2011)). Let p denote the number of proteins and n denote the number of samples. Simon *et al.*, 2011 show that the computational complexity for ridge regularized Cox model is $O(np)$, that is, for fixed n as p changes, or for fixed p as n varies, the computational time increases linearly in p or n.

Empirically, we calculated the computational time in minutes using 1 CPU with survival outcome (Table S4). The third and fourth columns below illustrate the computational time for the algorithm without or with ridge regularization, respectively. Because of the cross-validation and grid search, the computational time for the regularization method is longer than the Cox model. In a scenario similar to our data application, with a sample size of 400 and the number of mediators of 50, the computational times are around 1 minute and 12 minutes without and with using ridge regularization, respectively. If the number of mediators doubles to 100, the computational times are increased to 3 minutes and 24 minutes, respectively. The computational time can be shortened if we implement parallel computing techniques.

n	No. Med.	Weak Mediators Power	Strong Mediators Power
50	5	0.96	1.00
50	10	0.89	0.99
50	20	0.71	0.94
50	50	0.77	0.94
50	100	0.40	0.75
100	5	1.00	1.00
100	10	0.99	1.00
100	20	0.98	1.00
100	50	0.89	1.00
100	100	0.82	0.99
200	5	1.00	1.00
200	10	1.00	1.00
200	20	1.00	1.00
200	50	1.00	1.00
200	100	0.99	1.00
400	5	1.00	1.00
400	10	1.00	1.00
400	20	1.00	1.00
400	50	1.00	1.00
400	100	1.00	1.00
800	5	1.00	1.00
800	10	1.00	1.00
800	20	1.00	1.00
800	50	1.00	1.00
800	100	1.00	1.00

Table S3: Power for simulation with strong mediators and weak mediators using ridge penalties. The true indirect effect is -695 for the strong mediators, and the true indirect effect is -429 for the weak mediators.

**Sample size**	**No. mediators**	**Computational time in min. (no ridge)**	**Computational time in min. (with ridge)**
50	5	0.16	1.36
50	10	0.24	1.60
50	20	0.39	2.21
50	50	-	2.57
50	100	-	4.07
100	5	0.18	1.55
100	10	0.27	1.86
100	20	0.42	2.64
100	50	1.03	5.86
100	100	-	5.35
200	5	0.23	2.04
200	10	0.32	2.54
200	20	0.52	3.61
200	50	1.11	7.82
200	100	2.86	18.04
400	5	0.37	3.06
400	10	0.46	3.94
400	20	0.66	5.63
400	50	1.40	11.67
400	100	2.94	24.41
800	5	0.82	5.21
800	10	0.97	6.70
800	20	1.24	9.79
800	50	2.15	19.69
800	100	4.34	38.58

Table S4: Computational time in minutes using 1 CPU with survival outcome.

4 Proteogenomic Analyses in Kidney Cancer

4.1 TCGA-KIRC

4.1.1 Continuous exposures: mRNA

Pathway/Gene	mRNA
PTEN	PTEN
TCA cycle	CS, DLAT, DLD, DLST, FH, IDH1, IDH2, IDH3A, IDH3B, IDH3G, MDH1, MDH2, ACLY, ACO1, OGDH, ACO2, PC, PCK1, PCK2, PDHA1 ,PDHA2, PDHB, OGDHL, SDHA, SDHB, SDHC, SDHD, SUCLG2, SUCLG1, SUCLA2
Fatty acid synthesis	ACSF3, ACSL1, ACSL3, ACSL4, FASN, ACSBG1, ACSL6, MCAT, ACACA, ACACB, MECR, ACSL5, OXSM, OLAH, HSD17B8, ACSBG2, CBR4
AMPK	PRKAG2, PRKAG3, PRKAA1, PRKAA2, PRKAB1, PRKAB2, PRKAG1
Pentose phosphate	GLYCTK, FBP1, PRPS1L1, ALDOA, ALDOB, RPIA, ALDOC, G6PD, PGLS, GPI, C9orf103, DERA, PFKL, PFKM, PFKP, PGD, PGM1, PGM2, PRPS1, PRPS2, RPE, RBKS, TALDO1, TKT, LOC729020, TKTL1, TKTL2, FBP2, RGN, H6PD

Table S5: Key pathways and the gene members involved in metabolic shift for aggressive tumor in KIRC.

Outcome	Exposure	No. Exposure	No. Mediators
OS	PTEN	1	68
	TCA cycle	10	22
	Fatty acid synthesis	5	25
	AMPK Complex	2	25
	Pentose phosphate	12	15
PFI	PTEN	1	65
	TCA cycle	10	22
	Fatty acid synthesis	5	23
	AMPK Complex	2	31
	Pentose phosphate	11	16
DSS	PTEN	1	71
	TCA cycle	10	27
	Fatty acid synthesis	6	21
	AMPK Complex	2	36
	Pentose phosphate	12	22

Table S6: Number of the exposures (mRNA) and mediators (RPPA) for each of the survival outcomes in mediation analyses.

Pathway/gene	RNA	indirect[95%CI]	direct[95%CI]	total[95%CI]
PTEN	PTEN	-25.2 [-83.5;101.1]	96.4 [-27.3;193.9]	71.2 [-17.2;208.9]
TCA cycle	ACLY	49.6 [-9.4;113.3]	50.8 [-69.3;106.2]	100.5 [-30.6;165.3]
	ACO2	55.1 [-38.2;128]	-139.7 [-211.5;20.5]	-84.5 [-172.1;69.6]
	DLAT	38.8 [-15;157]	-42.4 [-216.5;96.6]	-3.6 [-149.8;160.5]
	IDH1	-20.2 [-143.4;-0.4]	-69.7 [-140.7;65]	-89.9 [-216.1;6.3]
	OGDH	-88.5 [-113.6;11.6]	100.1 [-5.1;180]	11.6 [-66.5;142]
	PCK2	-19.5 [-68.6;47.9]	68.9 [-35.3;133.7]	49.4 [-59.7;135]
	SDHB	-12.9 [-58;78.1]	15.5 [-106.9;98.1]	2.6 [-103.2;112]
	SDHD	-42.6 [-143.8;46.2]	52.4 [-144;165.7]	9.9 [-200.8;133]
	SUCLA2	7.2 [-15.7;114.4]	6.8 [-57.8;183.9]	14 [-10.5;226.5]
	SUCLG2	-9 [-82.1;61.7]	25.6 [-86.2;126.4]	16.6 [-115;122.9]
Fatty acid synthesis	ACACA	-17.3 [-75.7;54.6]	28.2 [-132.8;63.7]	10.9 [-140.7;54]
	ACSL1	2.5 [-31.8;84.8]	-25.7 [-72;90]	-23.1 [-55.8;118.1]
	CBR4	11.3 [-36;101.6]	-58.5 [-140.2;19.1]	-47.3 [-116.9;66.9]
	FASN	-93.8 [-150.4;-2.1]	-91.1 [-159.3;-10.4]	-185 [-260.7;-57.6]
	HSD17B8	49.7 [-13.5;106.8]	11.6 [-53;122.6]	61.4 [-11.2;169.2]
AMPK complex	PRKAA1	0.4 [-85.7;57.4]	22 [-85.5;94.7]	22.4 [-114.8;89.7]
	PRKAA2	103.4 [32.5;164.7]	65.2 [-30.6;144.8]	168.6 [76.8;228.5]
Pentose phosphate	C9orf103	7.8 [-45.9;65.5]	-2.7 [-108;65.8]	5.1 [-107;86.6]
	FBP1	42.3 [-14;74.6]	73.8 [-5.6;159.1]	116.1 [13.9;184]
	G6PD	-117.8 [-190.4;-29.7]	14.5 [-93.1;90.2]	-103.3 [-231.1;15.3]
	LOC729020	49.1 [-20.3;77.9]	21.8 [-59.8;90.6]	70.8 [-34.5;120.6]
	PFKP	17.7 [-48.1;55]	65.2 [-19.5;120.6]	82.9 [-17.2;125.6]
	PGLS	-16.9 [-63.2;62.9]	2 [-121.9;80.7]	-14.9 [-124.3;82]
	PGM2	13.9 [-32.6;74.3]	13 [-63.8;89.1]	26.9 [-54.8;122.1]
	RGN	18.5 [-62.6;41.4]	29.8 [-41.3;135.5]	48.3 [-61.2;129.4]
	RPIA	-19 [-99.6;24.1]	-1.3 [-76.6;60.1]	-20.3 [-131.8;39.7]
	TALDO1	5.4 [-51.5;70.7]	-22.8 [-87.5;91.6]	-17.4 [-91.2;101.8]
	TKT	-10.5 [-91.4;46.5]	-1.8 [-78.1;82.9]	-12.3 [-137.9;88.7]
	TKTL2	-12.1 [-63.8;44.6]	-31 [-97.3;48.5]	-43.1 [-117.7;49.8]

Table S7: Indirect, Direct, and Total effects and 95% confidence intervals (in days) of metabolomic mRNA expression as mediated by protein expression for OS

Pathway/gene	RNA	indirect[95%CI]	direct[95%CI]	total[95%CI]
PTEN	PTEN	-12.3 [-65.2;129.2]	42.3 [-82.1;145.3]	30 [-71.6;183.8]
TCA cycle	ACLY	54.2 [-40.6;94.7]	1.5 [-110.5;107.7]	55.7 [-80.7;144.3]
	ACO2	10.6 [-81;59.5]	-7.8 [-133.9;116.2]	2.7 [-164;118.9]
	DLAT	87.8 [-26.3;151.3]	20.2 [-143.2;159.3]	108 [-83.8;209.8]
	DLD	-71.8 [-169.7;27.1]	79.8 [-125.4;211.8]	8 [-202.5;153.8]
	DLST	14.9 [-58.8;109.7]	-38.3 [-122.4;86.7]	-23.4 [-107.6;118.3]
	IDH1	-75 [-149.5;8.3]	-77.5 [-196.6;42.9]	-152.5 [-271.2;-18.4]
	OGDH	7.2 [-71.9;69.9]	17 [-100.9;146]	24.2 [-106.6;155.9]
	PCK1	-6.1 [-45.7;48.5]	141.7 [24.2;215.4]	135.7 [22.1;226.3]
	SDHD	-9.9 [-75.4;167]	-117.3 [-309.6;51.5]	-127.1 [-259.3;89.9]
	SUCLA2	29.1 [-19.3;143.5]	26.4 [-141;218.3]	55.5 [-69.1;262.1]
Fatty acid synthesis	ACACA	-56.5 [-118.8;49.6]	14.1 [-76;126.2]	-42.4 [-121;87.9]
	ACSL1	10.2 [-39.6;85.3]	22.6 [-39.9;154.8]	32.8 [-20.8;176.7]
	CBR4	-7.5 [-35.4;78.3]	34 [-27.5;168.6]	26.5 [-2;191.3]
	FASN	-74.2 [-133.5;16.5]	-78.9 [-161.8;36.1]	-153.1 [-238.3;-19.6]
	HSD17B8	69.9 [-18.8;107.9]	50.1 [-59.5;133.4]	120 [-24.7;172.7]
AMPK complex	PRKAA1	34.1 [-48;122.5]	-65.5 [-159.7;55.7]	-31.4 [-128.6;97.5]
	PRKAA2	74.9 [-3.1;151.2]	124.8 [1.2;207.9]	199.7 [87.8;259.1]
Pentose phosphate	ALDOA	-0.4 [-88.8;23.3]	48.9 [-58.5;108.6]	48.4 [-100.8;92.8]
	C9orf103	19.4 [-60.5;60.4]	26.3 [-119.6;112.9]	45.7 [-126.3;120.7]
	FBP1	19 [-25.2;74.5]	104 [-32.1;210.5]	123.1 [-4.1;236.1]
	G6PD	-85.2 [-203.8;-17.8]	-12 [-199;95.2]	-97.2 [-307.9;2.2]
	LOC729020	-3.9 [-42.4;67.8]	25.8 [-70;115.4]	21.9 [-58.6;116]
	PGLS	-48.1 [-90.1;40.3]	-80.5 [-170.2;40.3]	-128.6 [-193.6;24]
	RBKS	-29 [-80.1;49.8]	-76.2 [-173.9;22.6]	-105.2 [-196.1;10.4]
	RGN	24.1 [-43.6;61.8]	23.9 [-73.7;141]	48 [-64.8;149.5]
	RPIA	-45.9 [-115.7;19.1]	-45.6 [-142.1;34]	-91.4 [-204.5;-6.1]
	TALDO1	35.8 [-22.8;104.6]	4.9 [-103;122.4]	40.7 [-73.7;158.8]
	TKT	21.8 [-80.5;68.7]	-7.2 [-64.6;163.3]	14.5 [-71.9;165.5]

Table S8: Indirect, Direct, and Total effects and 95% confidence intervals (in days) of metabolomic mRNA expression as mediated by protein expression for PFI

Pathway/gene	RNA	indirect[95%CI]	direct[95%CI]	total[95%CI]
PTEN	PTEN	21.7 [-94;88.4]	92.4 [-11.5;203.9]	114.1 [-37.6;202.9]
TCA cycle	ACLY	12.4 [-46.9;83.8]	33 [-72.9;102.1]	45.4 [-64.6;124]
	ACO2	-6.5 [-41.7;101.1]	-68.6 [-160.2;67]	-75.1 [-138.3;103.7]
	DLAT	51.2 [-30.9;97.3]	122.1 [-24.2;192.4]	173.3 [3.7;202.6]
	DLD	-46 [-127.3;79]	-44.3 [-258.5;111.3]	-90.3 [-285.4;70.9]
	IDH1	-47.1 [-139.7;40.5]	-65.5 [-158.2;56.9]	-112.6 [-209.8;24]
	OGDH	-11.6 [-71;51.7]	38.5 [-55.1;148.5]	26.9 [-71.5;147.1]
	PCK1	45.1 [-32.1;68.5]	-0.2 [-63.7;150.2]	44.9 [-54.2;157.7]
	PCK2	-30.7 [-76.5;26.3]	96 [-24.6;149.8]	65.3 [-54.1;140.6]
	SDHD	18.1 [-93.7;111.5]	-102 [-296.2;57.3]	-83.9 [-289.5;70.5]
	SUCLA2	42.7 [-22;115.7]	49.9 [-86;171.4]	92.7 [-30.2;191.4]
Fatty acid synthesis	ACACA	-15.2 [-62.9;57.5]	-12.3 [-118.4;28.3]	-27.6 [-138.6;48.7]
	ACSL1	-6.7 [-33.2;64.9]	49.3 [-36.1;123.3]	42.6 [-18.1;142.8]
	ACSL6	22.8 [-24.6;48]	137.2 [38;218.7]	160 [43.1;226.5]
	CBR4	38.8 [-32.6;80]	-28.1 [-87.7;68]	10.7 [-78.6;93.9]
	FASN	-45.2 [-137.1;11]	-52.4 [-137.4;3.7]	-97.6 [-227.4;-38.7]
	HSD17B8	68.6 [-4.7;122.8]	12.8 [-88.7;79.2]	81.4 [-42;131.1]
AMPK complex	PRKAA1	-13.7 [-76.6;74.6]	-28.7 [-94.8;102.2]	-42.4 [-115.5;106.5]
	PRKAA2	34.8 [15.4;144.1]	45.7 [-34.9;135.6]	80.5 [48.5;203.4]
Pentose phosphate	C9orf103	25.5 [-43.1;80.7]	-43.6 [-157;38.1]	-18.1 [-142.4;65.5]
	FBP1	23.5 [-20.2;79.5]	91.1 [-13.5;153.3]	114.6 [7.6;182.2]
	G6PD	-121.2 [-204;-20.7]	14.4 [-119.1;78.8]	-106.8 [-262.4;-5.9]
	GLYCTK	-25.9 [-81;39.7]	-18.4 [-124.8;30.5]	-44.3 [-158.9;26]
	LOC729020	22.8 [-37.1;75.1]	-33.6 [-83.4;75.5]	-10.9 [-69;99.3]
	PFKP	12.8 [-75.5;25.9]	74.1 [-9.3;121.7]	86.9 [-45;114]
	PGLS	-59 [-97;16.1]	43.6 [-74.7;97.3]	-15.4 [-133;66.5]
	RGN	-29.1 [-60.5;44.7]	-1.8 [-51.5;130.4]	-31 [-64.7;136.9]
	RPIA	-28.8 [-100.2;38.7]	-54.9 [-135.6;23]	-83.7 [-176.3;8]
	TALDO1	34.8 [-30.5;96.1]	2.9 [-99.5;90.6]	37.6 [-71.8;126.7]
	TKT	1.9 [-94.7;70.4]	28.2 [-70.5;106.5]	30 [-100.8;112.4]
	TKTL2	11.2 [-62.9;49]	-23.7 [-103.3;53.2]	-12.4 [-124.9;53.8]

Table S9: Indirect, Direct, and Total effects and 95% confidence intervals (in days) of metabolic mRNA expression as mediated by protein expression for DSS.

Pathway	Protein
Apoptosis	BAD, BCL2
Cell cycle	CCNB1, CDKN1B
EMT	SERPINE1, CTNNB1
Hormone signaling (Breast)	ESR1
PI3K/AKT	PTEN, CDKN1B, AKT1S1, TSC2, AKT1 AKT2 AKT3
RAS/MAPK	MAPK1 MAPK3, MAP2K1, RPS6KA1, YBX1
RTK	ERBB3, EGFR, SRC, ERBB2
TSC/mTOR	RB1, EIF4EBP1, RPS6, RPS6KB1, MTOR
Core reactive	CTNNB1
Breast reactive	CTNNB1, RAB11A RAB11B, MYC
Others	YWHAZ, ACACA ACACB, PRKAA1, ANXA7, ASNS, BRAF, KIT, DPP4, PECAM1, DVL3, EIF4G1, FASN, FOXO3, G6PD, GAB2, IGFR1, IGFBP2, IRS1, ERRFI1, MSH6, NDRG1, CDH3, PDK1, PEA15, PIK3R1, RPTOR, PTPN11, DIABLO, TFRC, XBP1

Table S10: Proteins significantly mediate the effect of the PTEN on PFI at $\alpha = 0.05$ with ridge penalty.

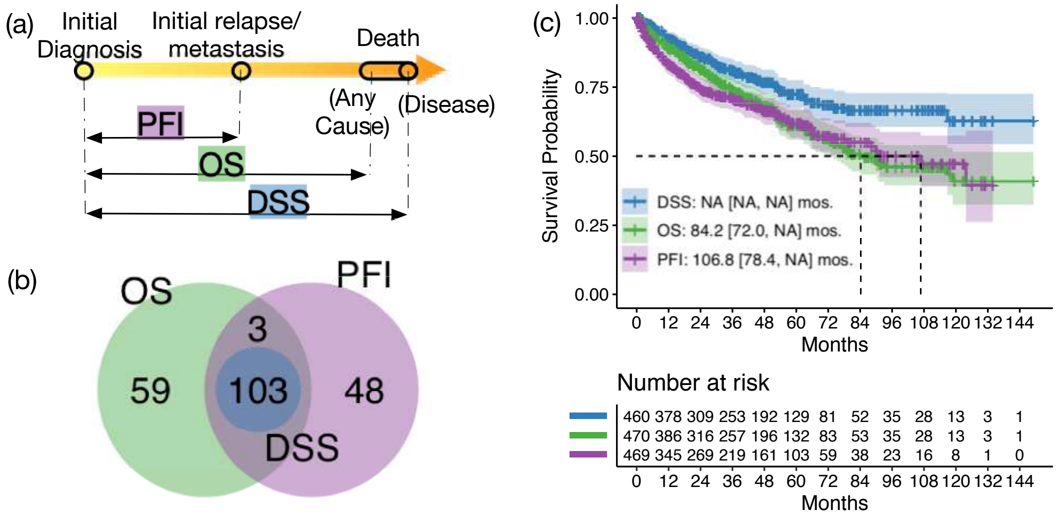

Figure S2: (a) Illustration of the definition of time to event for DSS, OS, and PFI. (b) Venn diagram of the number of events for DSS, OS, and PFI. (c) Kaplan-Meier Plots of DSS, OS, and PFI (in months), as well as their median survival and 95% confidence intervals.

4.1.2 Binary exposure: mutation

A mutation has the ability to regulate the level of protein expressions but could also alter proteins' function without altering the expression levels. Therefore, it is reasonable to view a mutation as a potential exposure and assess how much of its effect on survival is mediated by proteins. We conducted analyses for somatic mutations as exposures for all three types of survival outcomes. We included nine genes that were found to be highly mutated by TCGA Research Network (2013). Figure S3 illustrates the mutation frequencies of the selected genes within samples having the matched samples across mutation, RPPA, and clinical outcomes. The mutation frequencies range from 3% to 50%. VHL and PBRM1 genes are the top two mutations with frequencies more than 30%. After the prescreening procedure of the protein mediators as described in Section 4, the number of potential mediators across exposures ranges from 27 to 47 proteins.

Table S11 and Figure S4 summarize the indirect, direct, and total effects and 95% confidence intervals (in days) of a mutation as mediated by protein expression for PFI in TCGA-KIRC. BAP1 mutation has a significant total effect on OS, PFI, and DSS, which has the same direction as reported in TCGA Research Network (2013). It is associated with worse survival outcomes (Table S11). BAP1 also has a significant

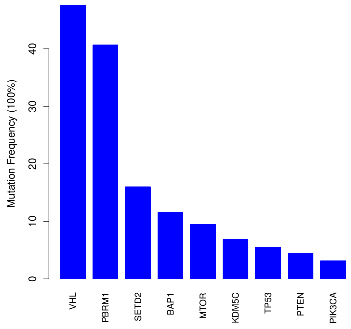

Figure S3: Barplot of mutation frequencies of nine highly mutated genes in KIRC reported by TCGA Research Network (2013)

indirect effect on OS and DSS. On average, BAP1 mutation has a total effect of reducing mean restricted lifetime in OS by 300 days, with 234 days explained by changes in protein mediators, 78% (234/300) of the total effect. Besides, It has a total effect of reducing mean restricted lifetime of DSS by 374 days, with 227 days explained by changes in protein mediators, 61% (227/374) of the total effect. Proteins that significantly mediate the mutation's effect on survival are summarized in Table S12.

Survival Outcome	Mutation	Indirect effect [95%CI]	Direct effect [95%CI]	Total effect[95%CI]
PFI Sample size: 337	VHL	28.8 [-78.2;151.5]	102.1 [-92.8;269]	130.9 [-59.9;312.2]
	PBRM1	153.7 [-54.2;356.6]	-270.5 [-544.4;-40.3]	-116.8 [-335.7;77.2]
	SETD2	-136.4 [-285.7;47.1]	-9.2 [-311.6;170]	-145.5 [-430.4;77.1]
	KDM5C	-0.7 [-226.8;229.3]	88.4 [-373.9;365.4]	87.7 [-332.3;357.5]
	BAP1	-196 [-418.3;41.7]	-222.3 [-570.5;52.3]	-418.3 [-751.5;-132.5]
	PTEN	-158.8 [-387.8;173.2]	82.6 [-300;378.5]	-76.2 [-485.8;340.3]
	MTOR	-143.3 [-356.6;47.7]	48.8 [-259.7;255.2]	-94.6 [-436.3;163.4]
	TP53	-205.8 [-617.7;109.5]	111.5 [-320.1;362.7]	-94.3 [-673.1;283.9]
	PIK3CA	91.6 [-237;347.4]	16.8 [-405.1;408.6]	108.4 [-313.1;416.3]
OS Sample size: 338	VHL	59.9 [-53.1;203.5]	-82.6 [-254.8;106.7]	-22.7 [-183.8;174.4]
	PBRM1	175.7 [37.9;359.8]	-125.6 [-419.8;8.3]	50.1 [-199.4;162.8]
	SETD2	-72.2 [-192.7;160.5]	-11.4 [-312.2;199.1]	-83.7 [-303.6;149]
	KDM5C	62.1 [-176.5;374.4]	-239.7 [-642.5;115.8]	-177.6 [-491.2;154.4]
	BAP1	-233.9 [-416.4;17.5]	-65.9 [-404.1;97.4]	-299.9 [-603.2;-69.3]
	PTEN	-97.9 [-375.2;152.2]	-4.8 [-393.8;301.7]	-102.7 [-518.1;286.7]
	MTOR	-153.8 [-339.4;52.1]	66.2 [-276.2;209.3]	-87.6 [-442.1;110.7]
	TP53	-247.9 [-701.9;37.6]	1.1 [-471.1;286.9]	-246.9 [-865.3;149.3]
	PIK3CA	3 [-227.8;163.1]	166.5 [-206.2;411.3]	169.5 [-228.9;417.9]
DSS Sample size: 332	VHL	92.3 [-79.3;192.2]	-1.5 [-155.2;240.9]	90.8 [-99.2;272.5]
	PBRM1	78.9 [-114.3;277]	-71.7 [-332.2;136.2]	7.2 [-224.9;185.7]
	SETD2	-101.6 [-288;153.8]	-87 [-364.4;121.8]	-188.6 [-418.8;49.9]
	KDM5C	80.3 [-190.5;436.4]	-170.3 [-598.6;194.8]	-90 [-433.7;234.7]
	BAP1	-227.2 [-398.9;102.3]	-147.5 [-514.4;94.7]	-374.7 [-594.9;-62.9]
	PTEN	-42.1 [-404.4;276.7]	10.7 [-354.8;289.2]	-31.3 [-450.9;299.9]
	MTOR	-114.5 [-348.8;131]	-86.4 [-454.3;176.8]	-200.9 [-541.7;76]
	TP53	-301.1 [-671.1;186.6]	-51 [-437.4;246.5]	-352.1 [-769.6;181.7]
	PIK3CA	-42.5 [-293.9;291.1]	149.2 [-437.2;359.2]	106.7 [-429.4;355.6]

Table S11: Indirect, Direct, and Total effects and 95% confidence intervals (in days) of a mutation on survival outcome as mediated by protein expression in TCGA-KRIC.

Outcome	Mutation	Significant RPPA
OS	BAP1	YWHAZ, ACACA ACACB, PRKAA1, AR, BAX, CTNNB1, CASP7, CCNB1, DVL3, FASN, GAB2, ERBB2, ERBB3, IGFR1, IGFBP2, MAPK1 MAPK3, MAP2K1, ERRFI1, MSH6, CDH3, CDKN1B, RPS6KA1, PDK1, PEA15, RPS6, PTPN11, SRC, TSC2, YBX1
	MTOR	YWHAZ, ACACA ACACB, PRKAA1, AR, ASNS, BAX, CTNNB1, CASP7, CCNB1, DVL3, FASN, GAB2, ERBB2, ERBB3, IGFR1, IGFBP2, MAPK1 MAPK3, ERRFI1, MSH6, CDH3, CDKN1B, RPS6KA1, SERPINE1, PEA15, RAB11A RAB11B, RPS6, PTPN11, SRC, SYK, TFRC, TSC2, YBX1
DSS	BAP1	YWHAB, YWHAZ, ACACA ACACB, PRKAA1, AR, BAX, CTNNB1, CASP7, PECAM1, CCNB1, DVL3, ESR1, FASN, GAB2, ERBB2, ERBB3, MAPK1 MAPK3, MAP2K1, ERRFI1, MSH6, CDH3, CDKN1B, RPS6KA1, SERPINE1, PDK1, PEA15, PIK3R1, RAB11A RAB11B, RB1, RPS6, PTPN11, DIABLO, SRC, TFRC, YBX1
	SETD2	YWHAZ, ACACA ACACB, PRKAA1, AR, ASNS, BAX, CTNNB1, CASP7, PECAM1, CCNB1, DVL3, ESR1, FASN, FOXO3, GAB2, ERBB2, ERBB3, IGFR1, MAPK1 MAPK3, MAP2K1, MSH6, CDKN1B, RPS6KB1, RPS6KA1, SERPINE1, PDK1, PEA15, PIK3R1, RAB11A RAB11B, RB1, RPS6, PTPN11, DIABLO, SRC, TFRC, YBX1

Table S12: Proteins significantly mediate the effect of the mutation on survival outcomes with ridge penalty in TCGA-KIRC

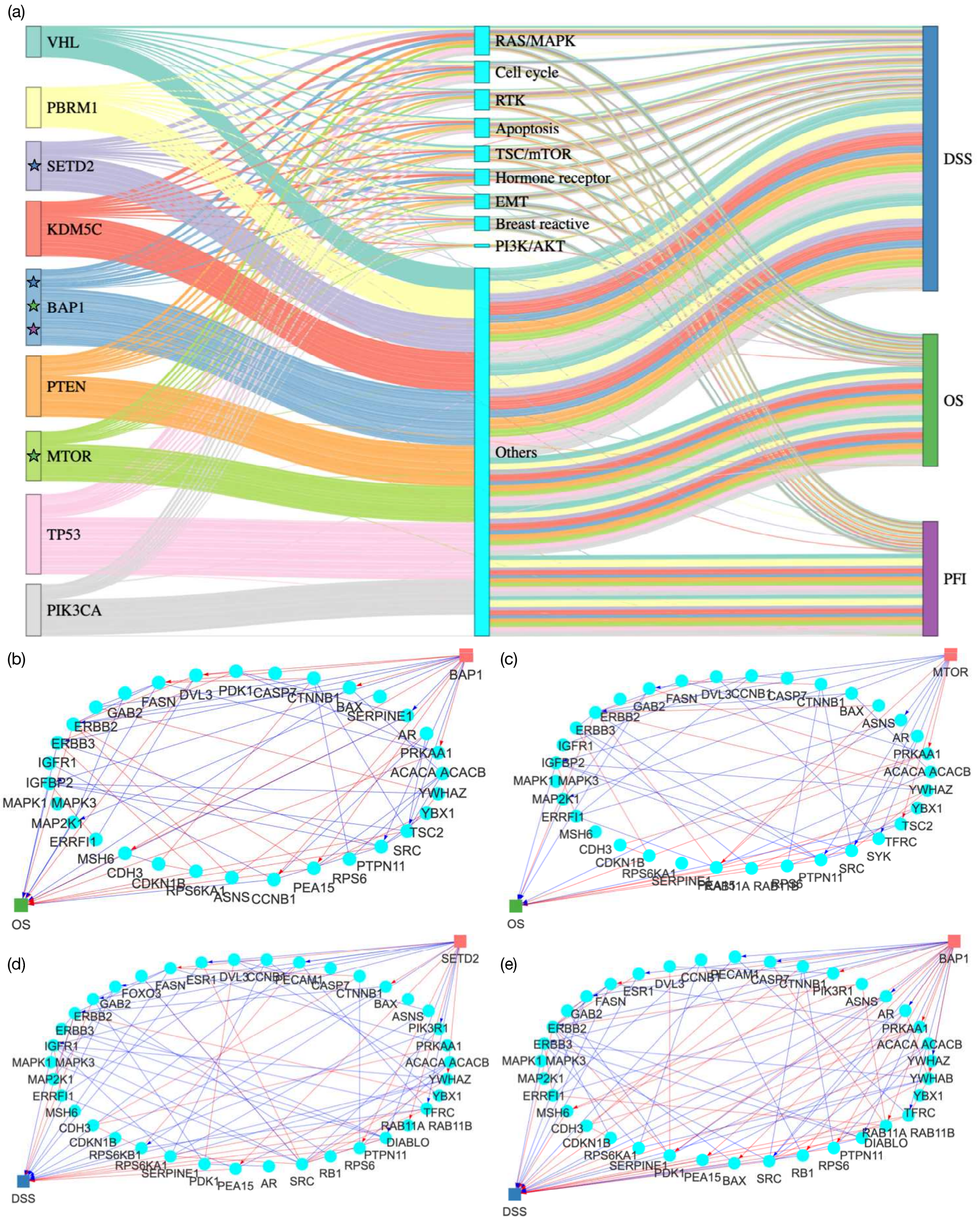

Figure S4: (a) Sankey Diagram illustrates the indirect and direct effects (in days) of mutation on three clinical survival outcomes as mediated by protein expressions (grouped into pathways). Nodes at the left are mutations, cyan nodes in the middle are proteins (grouped into protein pathways), and nodes at the right are three survival endpoints. Edges are color-coded by each of the mediation analyses with edge widths proportional to the estimated absolute value of coefficients in regression without ridge penalties. Significant results in total/direct/indirect effect with ridge penalties are highlighted with a star that is in the color that indicates the corresponding survival outcome. (b-e) Multilayered network of mutation on survival mediated by proteins. A path mutation → protein A → survival is connected if protein A is a significant mediator and the magnitude of the product of the path coefficients is larger than 0.05. Within proteins, we connect two proteins if the p-value of its partial correlation is less than 0.001. Red indicates positive coefficients, and blue indicates negative coefficients.

References

Efron, B. and Tibshirani, R. J. (1994). *An introduction to the bootstrap*. CRC press.

Network, T. R. (2013). Comprehensive molecular characterization of clear cell renal cell carcinoma. *Nature*, **499**(7456), 43–49.

Pearl, J. *et al.* (2009). Causal inference in statistics: An overview. *Statistics surveys*, **3**, 96–146.

Rubin, D. B. (1974). Estimating causal effects of treatments in randomized and nonrandomized studies. *Journal of educational Psychology*, **66**(5), 688.

Simon, N. *et al.* (2011). Regularization paths for cox's proportional hazards model via coordinate descent. *Journal of Statistical Software*, **39**(5), 1–13.

VanderWeele, T. J. and Vansteelandt, S. (2009). Conceptual issues concerning mediation, interventions and composition. *Statistics and its Interface*, **2**(4), 457–468.
